# Ethnobotanical biocultural diversity by rural communities in the Cuatrociénegas Valley, Coahuila; Mexico

**DOI:** 10.1186/s13002-021-00445-0

**Published:** 2021-03-29

**Authors:** Eduardo Estrada-Castillón, José Ángel Villarreal-Quintanilla, Juan Antonio Encina-Domínguez, Enrique Jurado-Ybarra, Luis Gerardo Cuéllar-Rodríguez, Patricio Garza-Zambrano, José Ramón Arévalo-Sierra, César Martín Cantú-Ayala, Wibke Himmelsbach, María Magdalena Salinas-Rodríguez, Tania Vianney Gutiérrez-Santillán

**Affiliations:** 1 Facultad de Ciencias Forestales, Universidad Autónoma de Nuevo León, Km 145 Carr. Nac. Linares-Cd. Victoria, A.P. 41, 67700 Linares, Nuevo Léon México; 2 Departamento de Botánica, Universidad Autónoma Agraria Antonio Narro, C.P. 25315, Buenavista, Saltillo, Coahuila México; 3 Capital Natural, A. C., Avenida Ricardo Margain Zozaya 440, Valle del Campestre, 66265 San Pedro Garza García, Nuevo León México; 4 Departamento de Botánica, Ecología y Fisiología Vegetal, Facultad de Ciencias, Universidad de La Laguna, San Cristóbal de La Laguna, Canary Islands Spain; 5 Herbario Jerzy Rzedowski, Facultad de Ciencias Naturales, Universidad Autónoma de Querétaro, Querétaro, 76221 México

## Abstract

**Background:**

Cuatrociénegas, part of the Chihuahuan Desert, is a region of unique biological, geological, geographical, and evolutionary importance. Its current population is mestizo; nevertheless, it has high national historical, cultural, and touristic relevance in Mexico. It has been cataloged as nationally significant for its flora and fauna by Mexican law, as well as being designated a High Protection site by the World Wildlife Fund and UNESCO. Because of its diverse and complex biological and sociocultural characteristics, we considered it important to determine, identify, and analyze various aspects of the traditional ethnobotanical knowledge and practices in this region.

**Methods:**

Between 2016 and 2019, seven field trips were made to document the knowledge and use of flora. Cuatrociénegas is a protected area, collecting botanical material is regulated, so specimens were photographed and collected in neighboring communities, and in public and private gardens. Later permission was obtained to complete the collection of specimens (2019–2020). The plants were identified and entered into the flora database of the state of Coahuila, and deposited in the Herbarium of the Faculty of Forest Sciences, Autonomous University of Nuevo León, Mexico. One hundred ten local residents (50 men and 60 women), aged between 27 and 91 years, were interviewed (semi-structured interviews). The cultural importance of ethnobotanical resources (cultural significance index) and its significance with respect to ethnobotanical richness in other Biosphere Reserves in Mexico (Mann-Whitney test), and similarities in the diversity of exotic species (Sørensen index) were studied.

**Results and discussion:**

The ethnobotanical information registers 158 species and 132 genera in 57 vascular and non-vascular families, documenting a greater knowledge and use of cultivated species (84) with respect to wild species (74). The diversity of plants reported is compared to other ethnobotanical studies carried out in Mexican Biosphere Reserves. These results are highly relevant, in spite of unique exotic species. The people local pay special attention to medicinal and ornamental plants. The species that presented the highest use values are *Larrea tridentata*, *Jatropha dioica*, and *Machaeranthera pinnatifida*, three species characteristic of the desert region.

**Conclusions:**

The particular diversity of wild flora in Cuatrociénegas Valley, combined with the varied introduced flora, is an important multifunctional resource. Special attention to introduced species is associated with harvesting use restrictions in the protected area as well as the high value of ornamental species that are difficult to maintain in desert areas. The extensive use of ethnobotanical knowledge is an example that biocultural diversity (at the conceptual level) is also strongly associated with socio-ecological systems incorporating mestizo groups and semi-urban rural landscapes, thus ceasing to be an exclusive focus of indigenous communities and regions.

**Supplementary Information:**

The online version contains supplementary material available at 10.1186/s13002-021-00445-0.

## Resumen

**Antecedentes:**

Cuatrociénegas es una región de singular importancia biológica, geológica, geográfica y evolutiva. Forma parte del Desierto de Chihuahua, su población actual es mestiza; sin embargo, posee una alta relevancia histórica, cultural y turística. Ha sido catalogado como Área de Protección de Flora y Fauna por la ley mexicana, así como un sitio de Alta Protección por el Fondo Mundial para la Naturaleza y la UNESCO. Por sus complejas características biológicas y socioculturales, consideramos importante conocer, determinar, identificar y analizar los conocimientos y prácticas etnobotánicas tradicionales en esta región.

**Métodos:**

Entre 2016 y 2019 se realizaron siete viajes de campo para documentar el conocimiento y uso de la flora. Cuatrociénegas es un área protegida, la recolección de material botánico está regulada, por lo que se fotografíaron y colectaron especímenes en comunidades aledañas, y en jardines públicos y privados. Posteriormente se obtuvo el permiso para completar la colección de ejemplares (2019-2020). Las plantas fueron identificadas e ingresadas a la base de datos de flora del estado de Coahuila, y depositadas en el Herbario de la Facultad de Ciencias Forestales de la Universidad Autónoma de Nuevo León, México. Se entrevistó a ciento diez vecinos (50 hombres y 60 mujeres) de entre 27 y 91 años (entrevistas semiestructuradas). Se evaluó la importancia cultural de los recursos etnobotánicos (índice de importancia cultural) y su significancia con respecto a la riqueza etnobotánica en otras Reservas de Biosfera en México (prueba de Mann-Whitney) y la similitud en la diversidad de especies exóticas (índice de Sørensen).

**Resultados y Discusión:**

La información etnobotánica registra 158 especies y 132 géneros en 57 familias vasculares y no vasculares, documentando un mayor conocimiento y uso de las especies cultivadas (84) con respecto a las silvestres (74). La diversidad de plantas reportada, en comparación con otros estudios etnobotánicos realizados en Reservas de la Biosfera, es altamente relevante, sin embargo, las especies exóticas son únicas. Los pobladores locales tienen una especial atención a las plantas medicinales y ornamentales. Las especies que presentaron mayores valores de uso son *Larrea tridentata*, *Jatropha dioica* y *Machaeranthera pinnatifida*, tres especies características de la región desértica.

**Conclusiones:**

La particular diversidad de flora silvestre del Valle de Cuatrociénegas, combinada con la variada flora introducida, es un importante recurso multifuncional. La especial atención a las especies introducidas está asociada a las restricciones de aprovechamiento; así como a la alta valoración de especies ornamentales, dificiles de mantener en las zonas desérticas. El amplio conocimiento y uso de la etnobotánica son un ejemplo, que la diversidad biocultural (a nivel conceptual) también, está fuertemente asociada socio-ecologicamente a grupos mestizos y paisajes rurales semiurbanos, dejando de ser exclusiva de las regiones indígenas.

## Background

Arid lands in Mexico cover 60% of its area. Mostly, they are concentrated in northern regions [1]. These areas harbor a rich flora adapted to these hostile low rainfall environments [2], which include portions of two major deserts, the Chihuahuan Desert (CHD), and the Sonoran Desert. Cuatrociénegas is a region recognized as a living laboratory by and for the world scientific community. This is due to its outstanding historic, biodiversity, geologic, geographic, and evolutionary components. These characteristics have facilitated the development of research in microbial genomics [3], metagenomics [4], genetic variation, diversity and speciation of fishes [5], virus evolution [6], paleoecology [7], paleoclimate [8], limnology [9], microbial endemism [4], endemic algae [10], speleogensis [11], stratigraphy [12], and flora and vegetation [13, 14]. Cuatrociénegas is one of the few places in the world where stromatolites live, organisms characterized by their antiquity of billions of years [15].

The Cuatrociénegas Basin qualifies as an environment so unique that it has been designated as an “*Área de Protección de Flora y Fauna*” (Flora and Fauna Protected Area) by the Mexican federal government. The area is administered by the Mexican agency SEMARNAT (Secretaría del Medio Ambiente y Recursos Naturales), and due to its outstanding biodiversity, Cuatrociénegas was declared a protected area in 1994 [16]. It is considered a high-priority site for conservation by the Nature Conservancy, the World Wildlife Fund, and UNESCO, and has been listed as a Wetland of International Importance by Ramsar.

The vegetation types described for the Cuatrociénegas Vallery corresponds mainly to rosetophyllous and microphyllous desert scrub, halophytic, aquatic, and semi-aquatic vegetation of the CHD. These are associated with a floristic diversity of approximately 840 species of vascular plants, of which 12 species are legislatively considered as species at risk within Mexico.

Cuatrociénegas is located in the center of the CHD. Historically, it is important as the birth place of Mexican president Venustiano Carranza (1917–1920) [17]. Economically, the area stands out for its alfalfa forage production and the growing of grapes for white and red wine. One of the most profitable activities is tourism, mainly in connection with multiple thermal pools scattered throughout the area. These are associated with salt accumulation, consisting mainly of sulfates resulting from high evaporation [18]. Because of its culture, architecture, and traditions, this city is included in the list of “Magical Towns.” These are places with unique attributes, including unique symbolism, authentic stories, important historical facts, and charming daily life, all of which means that these towns can enhance their economies by developing even stronger tourism sector.

Cuatrociénegas was founded approximately in 1760. Its actual population is completely mestizo, lacking indigenous populations. However, in the past, the Valley of Cuatrociénegas had been inhabited by nomadic Coahuiltecos and Borrados groups. In the context of this historical biocultural diversity [19], traditional rural communities house biocultural heritage that has been important in the conservation of biological diversity and in ecosystem services [20]. Biocultural diversity helps lend an understanding of human–nature relationships, not only in largely intact indigenous cultural areas but also in urban spaces [21], or in landscapes or semi-urban areas like Cuatrociénegas.

Based on the complex biological and sociocultural characteristics of the study area, we set the following objectives: (i) to collect knowledge from the residents regarding flora species and their uses, (ii) to determine the main type of uses people give for the species, (iii) to identify whether the main species used are native or exotic, (iv) to contribute to understanding of structural elements of biocultural diversity in traditional rural regions, and (v) to contribute to the dissemination of knowledge about traditional ethnobotanical uses as part of preserving the historical cultural heritage of natural resources in semi-arid areas of Mexico.

## Methods

### Study site

**Fig. 1:**
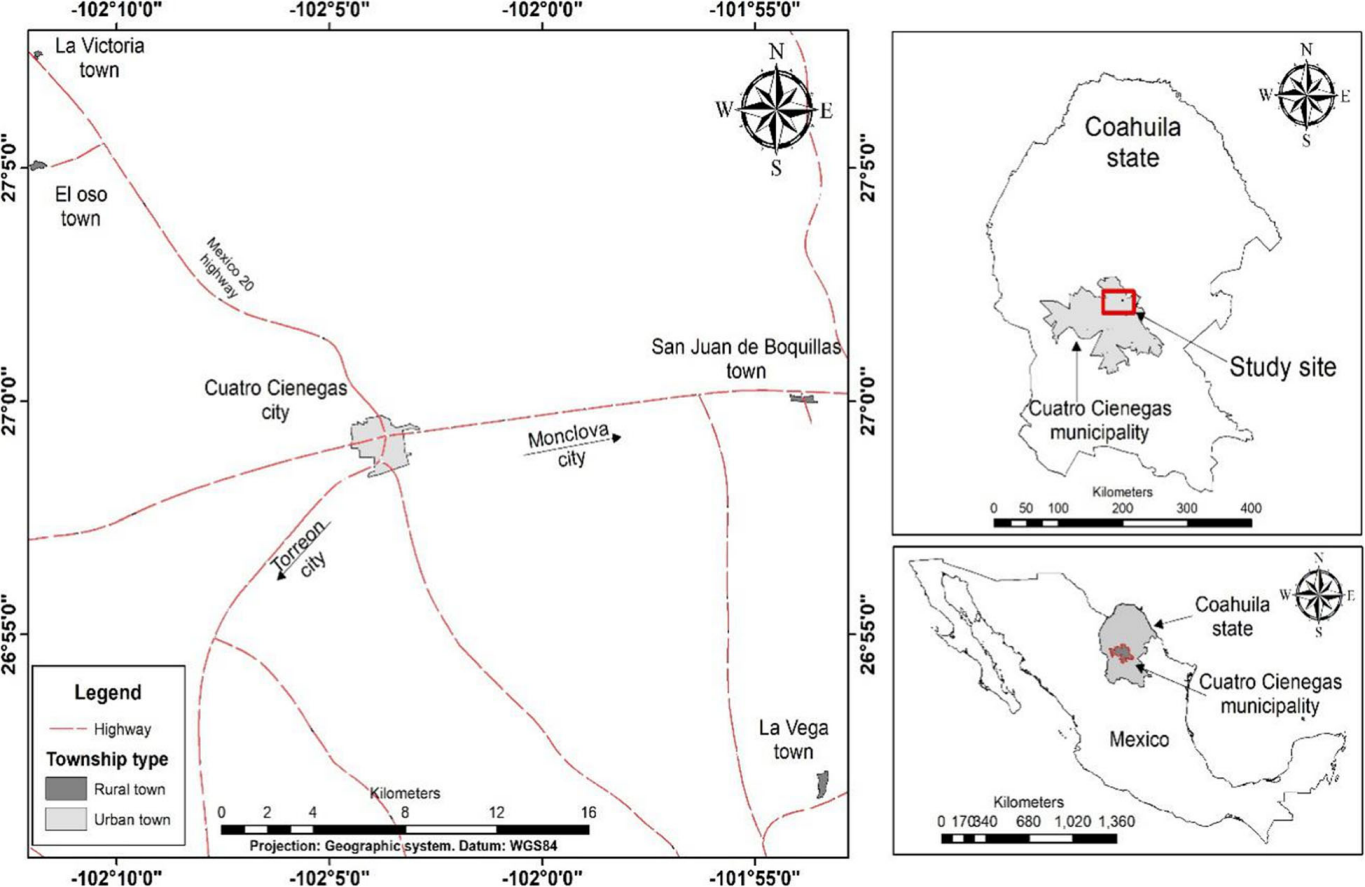
Geographic location of Cuatrociénegas, Coahuila, Mexico. Cuatrociénegas is an important region belonging to the Chihuahuan Desert. Due to its biological, geological, historical and cultural characteristics it is considered a “magical town”

Cuatrociénegas is a small city located in the central region of the state of Coahuila, 26° 42′ 10′′ to 26° 59′ 10′′ N, 101° 52′ 01′′ to 102° 03′ 59′′ W (Fig. 1). Its population is almost 13,000; it has all the modern services of elementary and basic education, social health care, and media and internet, and all the inhabitants speak Spanish. Physiographically, it is located in a valley at 740 m elevation, surrounded by high mountains reaching almost 2,900 m, which belong to the Sierra Madre Oriental range. Its climate is very dry; the most extreme temperatures in the valley reach 44 °C in summer, while in the mountains, the temperature falls below 0 °C in the winter. Annual precipitation is less than 200 mm [22]. Much of the water in the valley comes from groundwater, which emerges in the form of pools and springs; the landscape is characterized by its contrasting wet environments such as wetlands, marshes, underground streams, springs, rivers, lakes, temporary ponds, and groundwater [23]. According to its climate, geological, soil, water, and biological factors, it is considered one of the three most important desert ecoregions in the world [24].

### Vegetation and flora

Cuatrociénegas is part of the Chihuahuan Desert and its vegetation, like the flora and vegetation of arid environments, is essentially composed of dispersed shrub species [25]. Vegetation of the Cuatrociénegas Valley include rosetophyllous desert scrub, microphyllous desert scrub, halophytic vegetation, and aquatic and semiaquatic vegetation, with approximately 840 plant species [26], making up at least 25% of the flora of the state of Coahuila [27]. There are at least 70 species of endemic plants and animals in Cuatrociénegas [28].

### Ethnobotanical survey

In order to learn about the diversity of flora and its uses in the Cuatrociénegas region, seven field trips were carried out from 2016 to 2019 in order to photograph and record the plants, as well as to carry out ethnographic work. Since Valle de Cuatrociénegas is a protected area, collecting botanical material is regulated; initially, the identification of the plants was through the use of photographs by E. Estrada-Castillón and J.A. Villarreal-Quintanilla, based on a study of the flora of the state of Coahuila [27] and monographs of the genera distributed in this area. Plant specimens were collected from plant communities adjacent to the town, adjacent ejidos, and also from private and public gardens with the owners’ consent. Later, permission was obtained to complete the collection of plant specimens (2019–2020). The plants were identified and entered into the flora database of the Coahuila, as well as in the Herbarium of the Faculty of Forest Sciences, Autonomous University of Nuevo León, Mexico (the collection number belongs to the first author in Supplementary Material). Based on our experience in other studies, we decided to conduct the interviews by selecting people over 25 years of age or older. The ages of the interviewees ranged from 27 to 91. In order to ensure the reliability and homogeneity of the field information, all the interviewees who were selected were native-born or had lived there continuously for at least 25 years. The interviews were semi-structured in order to elicit the greatest amount of information in each interview and allow the free flow of information by the interviewees without limiting the free expression of ideas regarding species and uses. To this end, several key questions were included: What is the name of the plant? What do you use it for (medicinal, timber, food, fodder, seasoning, ritual)? How do you use it (raw, cooked, boiled, ground, battered)? What part of the plant do you use (root, stems, leaves, bracts, inflorescences, flowers, fruits, seeds, sap)? [29]. The interviews were conducted with the prior informed consent of each of the informants (International Society of Ethnobiology 2006; http://ethnobiology.net/codeofethics/ [30];). The informants were mainly homemakers, shepherds, and retirees, who knew the flora and its uses due to their custom of daily use of the various plants. During interviews were shown photographs of the species and plant specimens collected in private and public gardens and in areas adjacent to the protected area. We asked for the common names of the plants and the uses they are given (ethnographic technique of visual stimuli [29];). Informants were also asked about other wild and cultivated plants they knew and about the species they grew in their gardens. All information was recorded in Spanish, the only language spoken in the region.

### Data analysis

To compare the relevance of our study with respect to other ethnobotanical studies carried out in Biosphere Reserves in Mexico with mestizo and indigenous populations, a comparison was made using the Mann-Whitney test, calculated in the statistical program Past 3.20 [31]. The test was based on ethnobotanical data corresponding to the number of families, genera, and species; also included was information such as the extent of the reserve and the types of vegetation. The null hypothesis was rejected when the data of other reserves with respect to ours are similar; when the significance value of the test was less than *p* < 0.05. Obtaining information for comparison with other Biosphere Reserves in Mexico was carried out through a systematic review in electronic media, using a set of keywords (ethnobotany, protected natural area, Mexico, use, knowledge, plants [32]). On the other hand, to determine the similarity of the introduced species, the Sørensen index [31] was applied, comparing the exotic species with respect to other works in the northeast region and in Biosphere Reserves. Ethnobotanical data in these protected sites is scarce for the North region, especially the Northeast, where our study region is located. Therefore, the compared data are general and not specific to protected sites in areas belonging to the Chihuahuan Desert, where environmental conditions are similar.

In order to obtain and quantify the recorded information, the informant consensus factor (FIC [33];), fidelity level (FL [34];) and use value index (IVU [35];) were calculated. The FIC is an index that measures the relative importance of the different medicinal species for a category of use, and is calculated as FIC = *nur* – *nt*/*nur* − 1, where *nur* = number of uses mentioned, and *nt* = number of species used in each category. This quantitative technique enables the homogeneity of the information to be determined. Plants that are effective in treating certain diseases will have higher FIC. The fidelity level (FL) or Friedman index estimates the relative importance of each of the medicinal species based on the degree of consensus of the informants about the species’ use against a given category of use. With this index, the preferred species to cure certain diseases can be identified for certain informants; high FL values indicate that the medicinal species used to cure certain illness is widely used for that purpose. The fidelity level is calculated as FL(%) = *Ip*/*Iu*(100), where *Ip* = number of informants who independently indicated the use of a plant for the same particular illness; *Iu* = number of informants who mentioned the species for any illness within a category of use. The IVU is an index that quantifies the local importance of each of the species, and is calculated as IVU = ∑*Ui/n* where *Ui* = the number of uses mentioned by each informant for a given species, and *n* = the total number of informants.

## Results and discussion

### Ethnobotanical species diversity

According to the ethnobotanical information collected during interviews, the useful flora of Cuatrociénegas consists of 158 species in 132 genera of 57 vascular and non-vascular families (Table 1). Most of the species are herbaceous (68 species, 43.1%), followed by shrubs (53 species, 33.5%) and trees (37 species, 23.4%). Of the total species, 84 were introduced and 74 were native, which means that the inhabitants of Cuatrociénegas reported that they use more exotic than wild species. This low number of native species may be associated with the prohibition of collecting flora and fauna inside and around the periphery of the reserve. Therefore, the local people have a need to introduce ethnobotanical species that help satisfy their botanical needs, regardless of whether or not they are not locally native. In addition to having this greater appreciation for ornamental introduced species, these species are highly valued for their ease of acquisition and knowledge concerning their adaptations and cultural knowledge that facilitate maintenance. We assume that this ethnobotanical pattern is common in other desert regions of Mexico.

**Table 1:** Number of families, genera, and species of plants known and used by local residents of Cuatrociénegas, Coahuila, Mexico

	Eudicots	Monocots	Ferns and allies	Conifers	Total
Families	43	7	2	2	57
Genera	116	10	5	3	134
Species	138	11	5	5	159

The richness of ethnobotanical species in Cuatrociénegas is similar to that reported in other studies with mestizo communities, showing no significant differences with respect to knowledge and use of flora in Biosphere Reserves in Mexico (Table 2). For example, there is no significant difference between Cuatrociénegas and the ethnobotanical study in Cumbres de Monterrey National Park (*U*_d.f. 9_ = 11, n.s [36];), the Sierra de Huahutla Biosphere Reserve (*U*_d.f. 9_ = 12, n.s [37];), or the El Cielo Biosphere Reserve (*U*_d.f. 9_ = 11, n.s [38];). There is also no significant difference when the results are compared with an ethnobotanical study carried out in the Monarch Butterfly Biosphere Reserve with the Mazahua indigenous group (*U*_d.f. 9_ = 11, n.s [39];). It is important to note the often completely contrasting different types of ecosystems among the reserves; Cuatrociénegas corresponds to characteristics of the Chihuahuan Desert. However, it presents an environmental heterogeneity with different types of vegetation, thus in this sene, it is somewhat similar to Cumbres de Monterrey National Park, which may explain why there are no differences. However, when making comparisons with other Biosphere Reserves such as with Sierra de Huahutla, El Cielo, and even more homogeneous sites such as the Monarch Butterfly Biosphere Reserve, no differences are shown either, even when the territorial extension of the reserves has been taken into account (Table 2).

**Table 2:** Comparitive information for Biosphere Reserves in Mexico where ethnobotanical studies have been carried out. These data were obtained from publications [36–39] and from the catalog of Priority Terrestrial Regions for Mexico, CONABIO

	Ethnobotanical data		Extension (km2)	Vegetation types
Cuatrociénegas	Family	57	843	Halophilic, aquatic, and semi-aquatic vegetation, grassland, undergrowth scrub, submontane scrub, chaparral, pine, and oak forests
	Genus	132		
	Species	158		
El Cielo	Family	62	1445	Deciduous lowland forest, submontane scrub, cold forest, pine forest, medium sub-deciduous forest
	Genus	117		
	Species	69		
Cumbres de Monterrey	Family	69	4290	Pine forest, chaparral, submontane scrub, rosetophile desert scrub, oak forest, oyamel forest
	Genus	170		
	Species	240		
Sierra de Huahutla	Family	69	2959	Deciduous lowland forest, oak forest
	Genus	149		
	Species	185		
Monarch Butterfly	Family	66	4130	Pine forest
	Genus	142		
	Species	213		

However, in terms of the diversity and the ethnbotanical composition of Cuatrocienegas, and in particular the inclusion of exotic species, the data are unique compared to other sites in Northeast Mexico or Biosphere Reserves, thus showing low similarity [40]. For example, there is a high average similarity with the municipalities of Rayones (0.7663, [41]) and Bustamante (0.6391, [42]) in the neighboring state of Nuevo León. However, the dissimilarity is high compared to regions within the same state of Coahuila, despite maintaining similar ecological characteristics. But here, this dissimilarity occurs with different socio-cultural conditions. For example, with respect to the municipality of Muzquiz (0.3902 [43];), there are the only two indigenous settlements in northeastern Mexico, the Kikapo and the Mascogo blacks. Also, dissimilarity occurs with respect to the Lagunera region (0.1666 [44];), an area geographically close to Cuatrocienegas that has high levels of industrialization, and only some ethnobotanical reports in local markets where the exotic species would be expected to be similar (in terms of their commercialization). In addition, in comparison with the reported sites of the Biosphere Reserves, the similarity patterns remain low: (Cumbres de Monterrey [0.4285; 36]; Reserva de la Biosfera del Cielo [0.3703; 38]; Reserva de la Biosfera de la Mariposa Monarca [0.24; 39]; and Sierra de Huatla [0.1917; 37]). The high dissimilarities confirm that the ethnobotanical data for the Cuatrociénegas Biosphere Reserve are unique and that the exotic species used constitute a unique additional ethnobotanical diversity that seeks to maintain socio-ecological relationships. Therefore, the ethnobotanical richness in Cuatrociénegas is highly characteristic, and is as significant as in other studies, even in regions with the presence of indigenous groups [39]. It represents the importance of ethnobotanical resources in regions of northeast Mexico [36–38], and in desert areas.

Cuatrociénegas is an example of the use of ethnobotanical biocultural diversity in traditional mestizo rural regions as a means of cultural resilience. For this reason, the results acquire greater relevance, above all, if we consider that it is mentioned that indigenous groups protect and possess greater knowledge and relationships with nature. However, the history of occupation of a territory by mestizo peoples and their basic needs drive them to maintain a strong relationship between their population and the areas botanical resources. We can assume that ethnobotanical knowledge is not limited by the restrictions imposed in the management plans established in the reserve [16], to the sociocultural changes of semi-urban sites, to the cultural assignment, and even to the environmental characteristics of the region. This reaffirms that cognitive and pragmatic cultural niches are present in mestizo cultural baggage, allowing for the maintenance of socioecological systems.

**Fig. 2:**
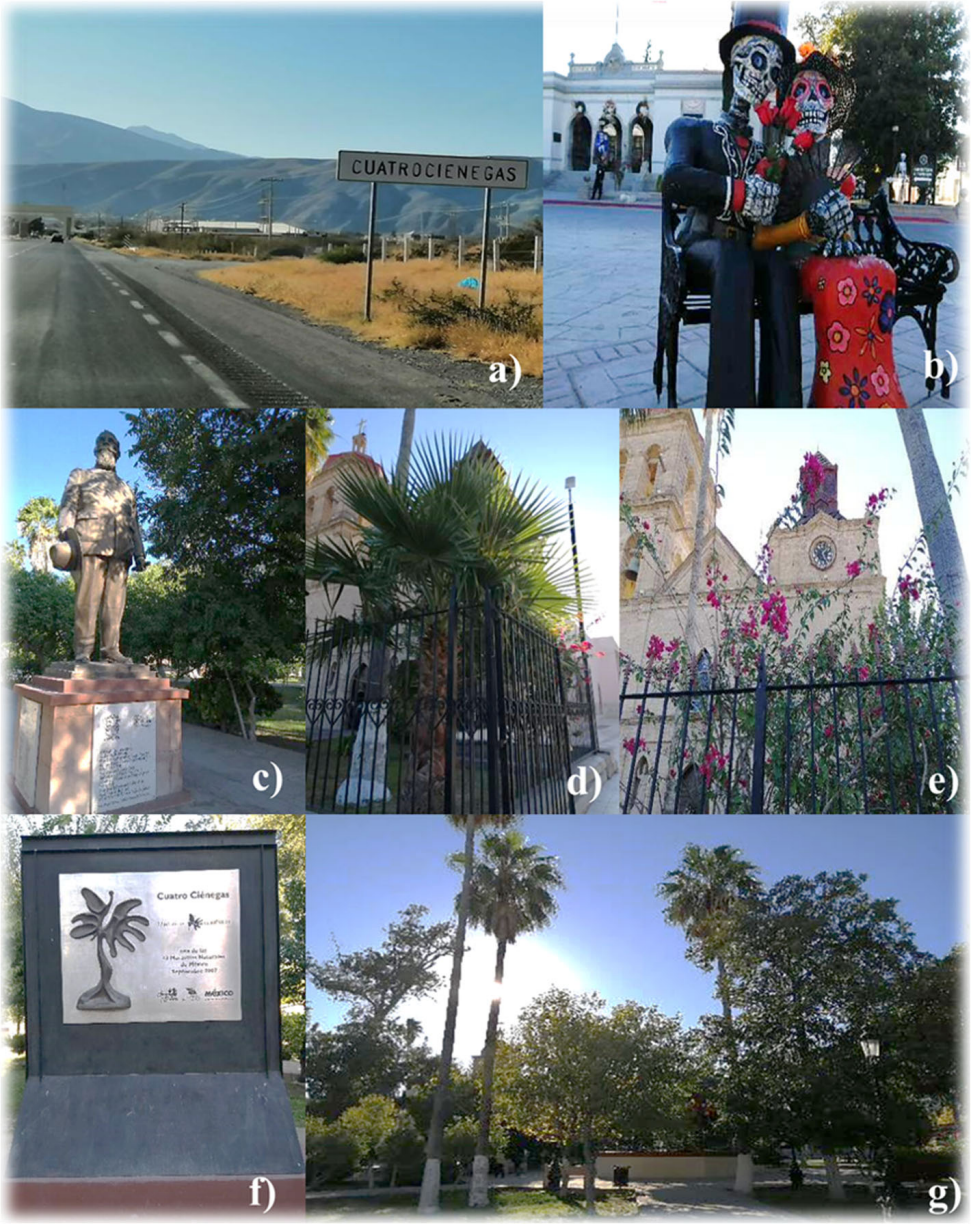
**a**–**g** Cuatrociénegas, Coahuila, Mexico: **a** general view of the entrance to the municipal seat; **b** Ciatrociénegas magical town, celebration of the day of the dead, November 2020; **c** statue in the central park of the illustrious president Venustiano Carranza, a historical figure; **d** *Brahea dulcis* and *Bougainvillea glabra*, ornamental elements in the atrium of the Parroquia of San José; **f** conmmemorative plaque “Cuatrociénegas 13 wonders of México,” Septembre 2007; **g** tree-lined in the central park of the municipal seat, ornamental elements. Author: Tania V. Gutiérrez-Santillán, Novembre 2020

This information should be taken into account for the redesign of plans for the conservation and management of the local flora in situ in the Cuatrociénegas reserve, considering the local inhabitants as direct actors in the conservation of floristic species. This should lead to generating avenues of action-participation between the government sector, academia, and local people. It is recommended through environmental education strategies, (a) the dissemination of botanical diversity, its knowledge and ethnobotanical applications; (b) the organization of informative action-participation workshops for the identification of native species and the recognition of their biological conservation status; (c) as well as the formation of groups of local producers of plants native and/or cultivated of cultural importance. This would translate into the revaluation and maintenance of ethnobotanical knowledge, sustainable economic opportunities for local people, and greater success in conservation of the characteristic and unique flora of Cuatrociénegas (Fig. 2a–g).

More species were native (95) than exotic (63; Table 3; Supplementary Material). Within the native species, a total of 21 (22%) cultivated species were registered. Several of these species are economically profitable, such as *Phaseolus vulgaris*, *Carya illinoinensis*, *Persea americana*, *Zea may*s, and *Solanum lycopseriscon* in northeastern Mexico [36, 40, 41]. Compared to the useful flora from four different areas, Cumbres de Monterrey National Park (240 species, 170 genera, 69 families [36];), Southern Nuevo León (163 species, 136 genera, 58 families [40];), Rayones (252 species, 228 genera, 91 families [41];), and Bustamante (218 species, 176 genera, 66 families [42];) in the adjacent state, Nuevo León, Cuatrociénegas, has a lower diversity of all taxa.

**Table 3:** Plant families, genera, species, and their uses in Cuatrocienegas, Coahuila, Mexico. The letter after author name indicates: *N* native, *E* exotic. The collection number belongs to the first author. The complete data of the descripction of the uses and their forms can be consulted in the Supplementary Material. Ornamental (I), Medicinal (II), Condment (III), Food (IV), Forage (V), Fibers (VI), Construction (VII), Fuel (VIII), Craft (IX), Liquor (X), Religious rites (XI), Fright (XII), Industrial (XIII), Wine industry (XIV), Furniture (XV)

Scientific name	Common name	Uses
**ACANTHACEAE**		
*Beloperone gutatta* Brendegee, N, 24957	Camarón	I
**ADOXACEAE**		
*Sambucus nigra* L., N, 24988	?	I, II
**ALLIACEAE**		
*Allium cepa* L., E, 24956	Cebolla	II, III
*Allium sativum* L., E, 25039	Ajo	II, III
**AMARANTHACEAE**		
*Amaranthus blitoides* S. Watson, N, 24968	Quelite	IV
*Atriplex canescens* (Pursh) Nutt., N, 25040	Chamizo, Costilla de vaca	V
*Celosia crista* L., N, 25000	Mano de león	I, II
*Dysphania ambrosioides* (L.) Mosyakin & Clemants, N, 24969	?	II, IV
*Spinacia oleracea* L., E, 25060	Espinaca	IV
**ANACARDIACEAE**		
*Schinus molle* L., E, 24955	Pirúl	I, XI, XII
**APIACEAE**		
*Coriandrum sativum* L., E, 24958	Cilantro	II, III
*Cuminum cyminum* L., E, 25061	Comino	III
*Daucus carota* L., E	Zanahoria	IV
*Petroselinum crispum* (Mill.) Fuss, E, 25062	Perejil	III
**APOCYNACEAE**		
*Nerium oleander* L., E, 25100	Laurel	I
*Cascabela thevetia* (L.) Lippold, E, 24954	?	I
*Vinca minor* L., E, 25099	Teresita	I
**ARECACEAE**		
*Washingtonia robusta* H. Wendl., N, 25001	Palma	I
**ASPARAGACEAE**		
*Agave lechuguilla* Torr., N, 25038	Lechuguilla, Amole	II, IX
*Agave parrasana* A. Berger, N, 25098	Maguey	I, III, IV, X
*Agave scabra* Ortega, N, 25063	Maguey	I, II, IV
*Dasylirion cedrosanum* Trel., N, 25002	Sotol	I, VI, X
*Sansevieria thyrsiflora* (Petagna) Thunb., E, 24970	Lengua de suegra, Guaco	I, II
*Yucca torreyi* Shafer, N, 25037	Palma	I, IV
*Yucca trecuelana* Carriere, N, 24971	Palma	I, IV
**ASTERACEAE**		
*Ageratina havanensis* (Kunth) R.M. King & H. Rob., N, 25036	Ageratina	I
*Artemisia ludoviciana* Nutt., N, 25097	Estafiate	II
*Calendula officinalis* L., E, 24972	Cartulina	I, II
*Chrysactinia mexicana* A. Gray, N, 24987	Hierba de San Nicolás	II
*Cynara scolymus* L., E, 24959	Alcachofa	II
*Flourensia cernua* DC., N. 25035	Hojasé	II
*Gnaphalium semiamplexicaule* DC., N, 25064	Gordolobo	II
*Lactuca sativa* L., E, 24960	Lechuga	IV
*Machaeranthera pinnatifida* (Hook.) Shinners, N, 25065	Árnica	II
*Matricaria chamomilla* L., E, 25066	Manzanilla	II
*Parthenium argentatum* A. Gray, N, 25095	Guayule	I
*Parthenium incanum* Kunth, N, 25094	Guayule	I, II
*Tagetes erecta* L., N, 24973	Cempazuchitl	I
**BIGNONIACEAE**		
*Chilopsis linearis* (Cav.) Sweet, N, 24986	Mimbre	I
*Tecoma stans* (L.) Juss. ex Kunth, N, 25034	San Pedro	I
**BORAGINACEAE**		
*Symphytum officinale* L., E, 24974	Suelda	I, II
*Beta vulgaris* L., E, 24961	Betabel	IV
*Raphanus sativus* L, E, 25033	Rábano	III, IV
**CACTACEAE**		
*Ariocarpus fissuratus* (Engelm.) K. Schum., N, 25091	Chaute	I, II
*Coryphantha pseudoechinus* Boed., N, 25092	Chilitos	I
*Cylindropuntia leptocaulis* (DC.) F.M. Knuth, N, 24985	Tasajillo	I, II
*Cylindropuntia imbricata* (Haw.) F.M. Knuth, N	Cardenche	I
*Echinocactus horizonthalonius* Lem., N, 25093	Manca caballo	I, II
*Echinocactus platyacanthus* Link & Otto, N, 24989	Biznaga burra	I, II, IV, V
*Echinocactus texensis* Hoppfer, N, 25032	Manca caballo	I, II
*Echinocereus enneacanthus* Engelm., N, 25090	Pithaya	II, IV, V
*Echinocereus pectinatus* (Scheidw.) Engelm., N, 25051	Pithaya	I, II, V
*Epithelantha micromeris* (Engelm.) Weber, N, 25067	Biznaguita blanca	I, II
*Ferocactus pilosus (Engelm.)* F.A.C. Weber ex Britton & Rose, N, 24990	Barril de fuego	I, II, IV, V,
*Lophophora williamsii* (Lam. ex Salm-Dyck) J.M. Coult., N, 25030	Peyote	I, II
*Opuntia engelmannii* Salm- Dyck ex Engelm., N, 25003	Nopal	II, IV, V
*Opuntia ficus-indica* (L.) Mill., N, 25070	Nopal criollo	I, II, V
*Opuntia grahamii* Engelm., N, 25004	Nopal	II, V
*Opuntia imbricata* (Haw.) DC., N. 25029	Coyonoxtle	II
*Opuntia phaeacantha* Engelm., N, 25028	Nopal rastrero	II, V
**CANNABACEAE**		
*Celtis pallida* Torr., N, 25089	Granjeno	IV
**CAPRIFOLIACEAE**		
*Lonicera japonica* Thunb., N, 25005	Madreselva	I
**CARCIACEAE**		
*Carica papaya* L., N, 24962	Papaya	I, II, IV
**CASUARINACEAE**		
*Casuarina cunninghamiana* Mig., E, 25027	Casuarina	I, VII
**CUCURBITACEAE**		
*Cucurbita pepo* L., N, 25068	Calabaza	IV
*Ibervillea sonorae* (S.Watson) Greene, N, 24963	Wereke	II
*Citrullus lanatus* (Thunb.) Matsum. & Nakai, E	Sandía	IV
*Cucumis melo* L., E, 25026	Melón	IV
*Cupressus arizonica* Greene, N, 25006	Ciprés	I, VIII
*Cupressus sempervirens* L., E, 25007	Pincel	I, VIII
*Juniperus flaccida* Schltdl., N, 25008	Táscate	I, VIII
*Arctostaphylos pungens* Kunth, N, 25101	Pingüica	II
*Cnidoscolus aconitifolius* (Mill.) I.M. Johnst., N, 25025	Chaya	I, II
*Croton suaveolens* Torr., N, 25087	Salvia	II
*Euphorbia antisyphilitica* Zucc., N, 25088	Candelilla	XIII
*Jatropha dioica* Sessé, N, 25059	Sangre de drago	II
*Ricinus communis* L., E, 25057	Higuerilla	II
*Tragia ramosa* Torr., N, 25058	Mala mujer	II
**FABACEAE**		
*Acacia farnesiana* (L.) Willd., N, 24991	Huizache	II, V, VII
*Caesalpinia mexicana* A. Gray, N, 25102	Hierba del potro	I
*Dalea bicolor* Willd., N, 24992	Engorda cabras	V
*Eysenhardtia texana* Scheele, N, 25056	Vara dulce	V
*Phaseolus vulgaris* L., N	Frijol	IV
*Prosopis glandulosa* Torr., N, 25103	Mezquite	IV, V, VII
*Vicia faba* L., E, 25024	Haba	IV
**FAGACEAE**		
*Quercus* spp., N, 25055	Encino	VII
**FOUQUIERIACEAE**		
*Fouquieria splendens* Engelm., N. 25069	Albarda, Ocotillo	VII
**GERANIACEAE**		
*Pelargonium zonale* (L.) L’Hér. ex Aiton, E, 25023	Geranio	I
**JUGLANDACEAE**		
*Carya illinoinensis* (Wangerin) K. Koch, N, 25054	Nogal	II, IV
*Juglans microcarpa* Berl., N	Nogalillo	VII
*Juglans major* (Torr.) Heller, N, 24993	Nogal de nuez Encapsulada	IV
**LAMIACEAE**		
*Hedeoma costata* Hemsl., N, 25104	Poleo	II, III
*Majorana hortensis* Moench, E, 24964	Mejorana	II, III
*Marrubium vulgare* L., E, 25086	Marrubio	II
*Melissa officinalis* L., E, 24975	Toronjil	II, IV
*Mentha* x *piperita* L., E, 24976	Yerbabuena	II, III
*Mentha spicata* L., E, 25022	Yerbabuena	II, III
*Ocimum basilicum* L., E, 24994	Albahaca	II, III, IV
*Poliomintha glabrescens* A.Gray ex Hemsl., N, 25053	Orégano	II, III
*Rosmarinus officinalis* L., E, 25021	Romero	II, III
*Salvia officinalis* L., E, 25020	Salvia real	II, III
*Thymus vulgaris* L., E, 24965	Tomillo	II, III
**LAURACEAE**		
*Cinnamomum verum* J. Presl., E, 25009	Canela	III
*Litsea pringlei* Bartlett, N, 24995	Laurel	II
*Persea americana* Mill., N, 24978	Aguacate	IV
*Persea americana* Mill. Var *drymifolia* (Schltdl. & Cham.) S. F. Blake, N, 24977	Aguacate criollo	II
**LILIACEAE**		
*Asparagus officinalis* L., E, 25010	Aspárago	I
**LYTHRACEAE**		
*Punica granatum* L., E, 24979	Granada	II, IV
**MALVACEAE**		
*Gossypium hirsutum* L., N, 24996	Algodón	I, VI
*Hibiscus rosa-sinensis* L., E, 24966	Hibisco	I
*Hibiscus syriacus* L., E, 25011	Rosa de Siria	I
**MELIACEAE**		
*Melia azedarach* L., 24967	Canelón lila	I, VIII
**MONIMIACEAE**		
*Peumus boldus*, E, 25019	Boldo	I, II
**MORACEAE**		
*Ficus carica* L., E, 25012	Higuera	I, IV
*Morus celtidifolia* Kunth, N, 25085	Mora	I, IV
**MORINGACEAE**		
*Moringa oleifera* Lam., E, 25071	Moringa	I, II
**MYRTACEAE**		
*Eucalyptus camaldulensis* Dehnh., E, 25018	Eucalipto	II, VIII
*Eucalyptus globulus* Labill., E, 25072	Eucalipto	II, VIII
**NYCTAGINACEAE**		
*Bougainvillea glabra* Choisy, N, 25105	Bugambilia	I, II
*Mirablis jalapa* L., N, 25052	Maravilla	I
**OLEACEAE**		
*Fraxinus americana* L, N, 25017	Fresno	I, VII
*Ligustrum japonicum* Thunb, E, 25051	Trueno	I
*Olea europea* L, E, 25106	Olivo	I
**PINACEAE**		
*Pinus cembroides* Zucc., N, 25049	Pino piñonero	I, IV, VII
*Pinus pinceana* Gordon, N, 25050	Pino	I, VII
**PLATANACEAE**		
*Platanus occidentalis* L., N, 25107	Álamo	I
**POACEAE**		
*Arundo donax* L., E, 25108	Carrizo	VII
*Avena sativa* L., E, 25109	Avena	II, IV, V
*Hordeum vulgare* L., E, 25048	Cebada	V
*Sorghum bicolor* (L.) Moench, E	Sorgo	V
*Zea mays* L., N, 25047	Maíz	II, IV, V
**PORTULACACEAE**		
*Potrulaca oleracea* L., N, 25073	Verdolaga	II
**PTERIDACEAE**		
*Adiantum capillus-veneris* L., N, 25110	Culantrillo	I
*Argyrochosma limitanea* (Maxon) Windham, N, 25074	Helecho	I
*Asplenium exiguum* Bedd., N, 25046	Helecho	I
*Pleopeltis guttata* (Maxon) E.G. Andrews & Windham, N	Helecho	I
**RHAMNACEAE**		
*Ziziphus jujuba* Mill., E, 25084	Jujube	I, IV
**ROSACEAE**		
*Cydonia oblonga* Mill., E, 25083	Membrillo	I, II, IV
*Eriobotrya japonica* (Thunb.) Lindl., E, 24980	Níspero	I, IV
*Prunus armeniaca* L., E, 25045	Chabacano	I, IV
*Prunus domestica* L., E, 25082	Ciruelo	I, IV
*Prunus persica* (l.) Batsch, E, 25081	Durazno	I, IV
*Rosa gallica* L., E, 25014	Rosa	I
*Rosa* sp., E, 25013	Rosa	I
**RUTACEAE**		
*Citrus limon* (L.) Osbeck, E, 24982	Limón	I, II, IV
*Citrus* x *sinensis* (L.) Osbeck, E, 24981	Naranja	I, II, IV
*Ruta graveolens* L., E, 24983	Ruda	II, III
**SALICACEAE**		
*Populus alba* L., N, 25015	Álamo	I
*Salix nigra* Marshall, N,2 5016	Sauce	I
**SAPINDACEAE**		
*Aesculus hippocastanum* L., E, 25044	Castaño de la Indias	I, II
**SAURURACEAE**		
*Anemopsis californica* (Nutt.) Hook. & Anr., N, 24997	Hierba del manso	II
**SCROPHULARIACEAE**		
*Leucophyllum frutescens* (Berland.) I.M. Johnston, N, 25075	Cenizo	I, II
*Leucophyllum minus* A. Gray, N, 25080	Cenizo	II
**SELAGINELLACEAE**		
*Selaginella lepidophylla* (Hook. & Grev.) Spring, N, 24998	Flor de peña	II
**SOLANACEAE**		
*Capsicum annuum* L., N, 25043	Chile piquín	II, III
*Lycopersicon esculentum* Mill., N, 25111	Tomate	IV
*Solanum tuberosum* L., E, 25079	Papa	IV
*Solanum rostratum* Dunal, N, 25112	Mula	II
**TURNERACEAE**		
*Turnera diffusa* Willd. ex Schult., N, 25076	Oreganillo	II
**URTICACEAE**		
*Urtica chamaedryoides* Pursh, N, 25077	Ortiga	II
**VERBENACEAE**		
*Lippia graveolens* Kunth, N, 25042	Orégano	II, III
**VITACEAE**		
*Vitis vinífera* L., E, 25078	Uva	IV, XIV
**XANTHORRHOEACEAE**		
*Aloe vera* L., E, 25041	Aloe, Sábila	II, IV
**ZYGOPHYLLACEAE**		
*Larrea tridentata* (Sessé & Moc. ex DC.) Coville, N, 2499	Gobernadora	II, IX, XV
**ZYNGIBERACEAE**		
*Zingiber officinale* Roscoe, E, 24984	Jengibre	II

**Fig. 3:**
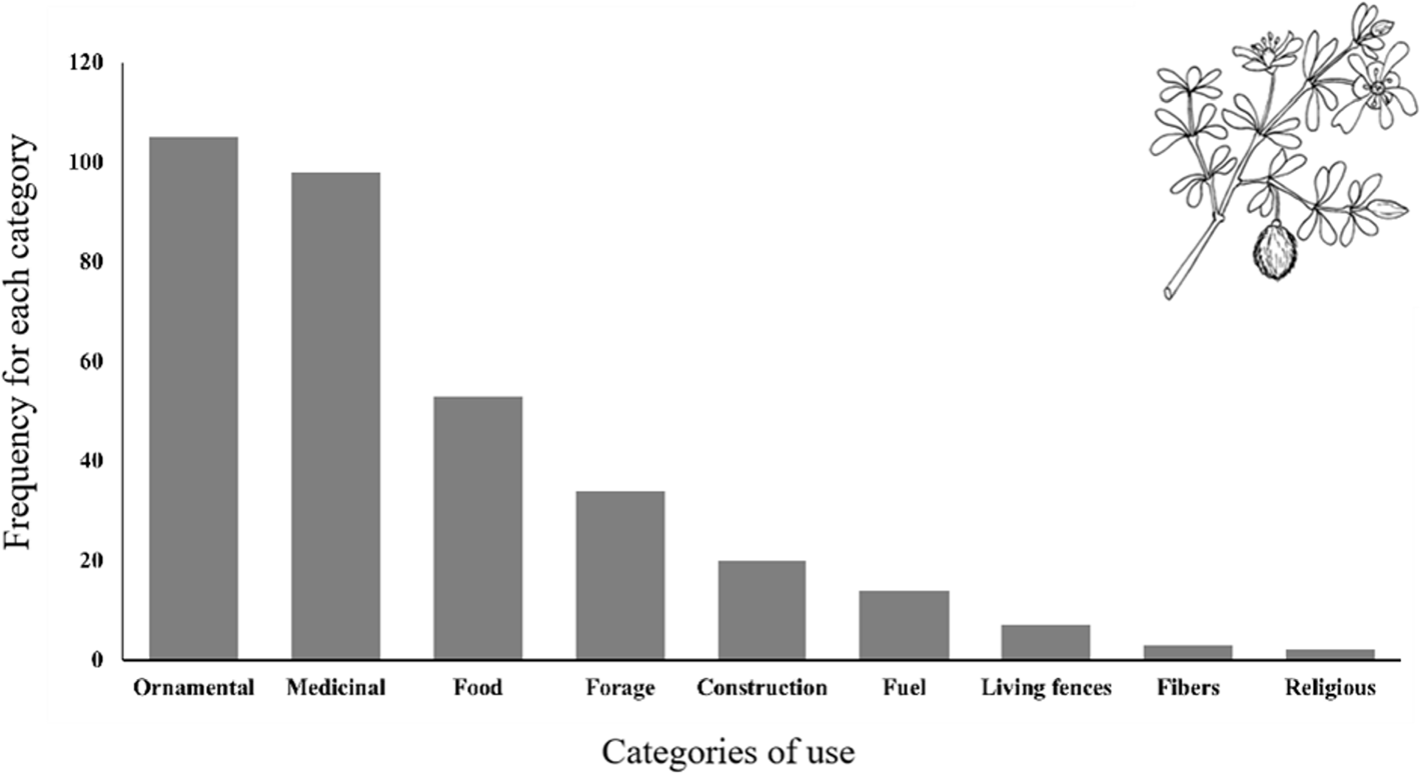
Number of plant species mentioned by the interviewees, organized by use categories

**Fig. 4:**
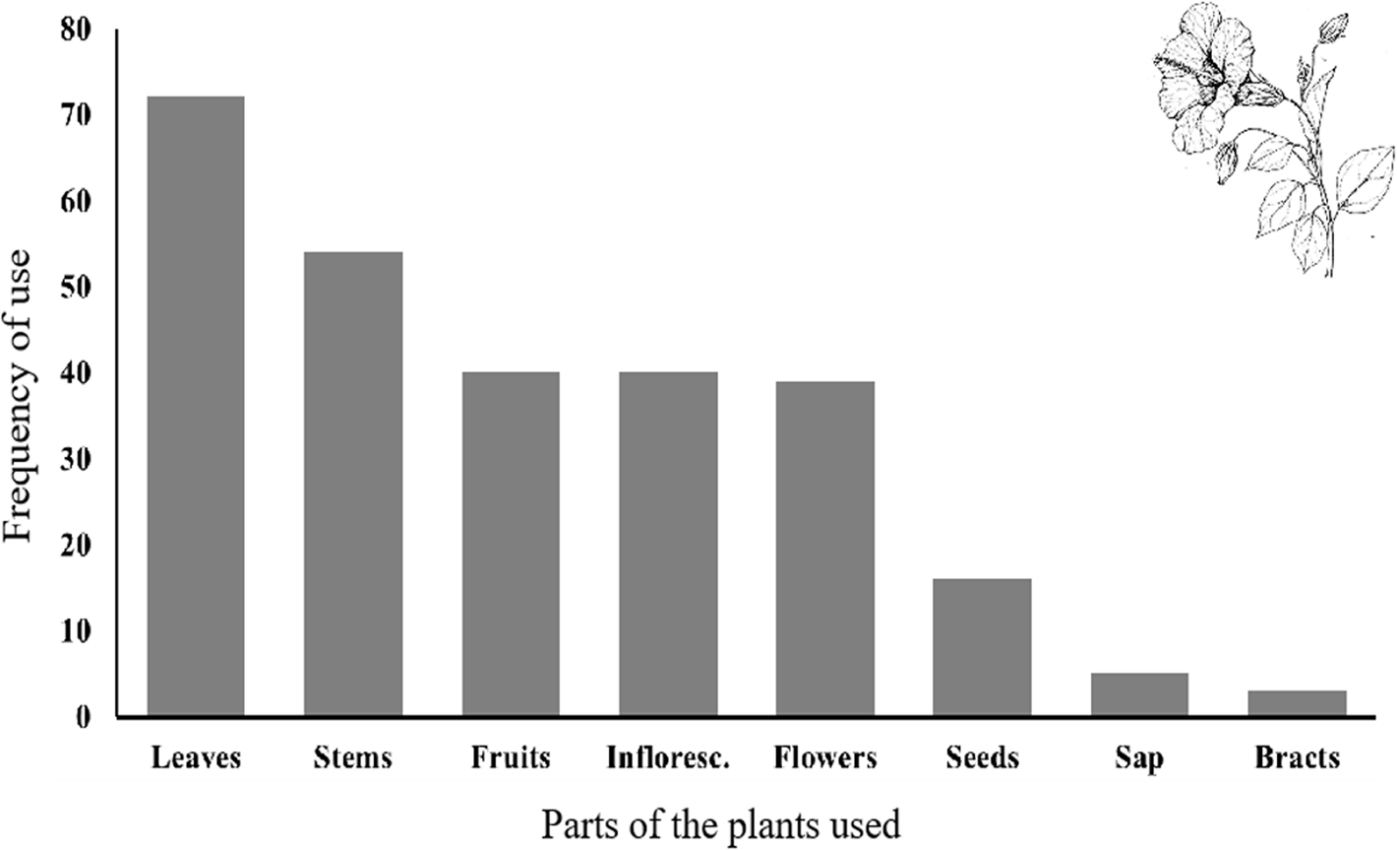
Parts of the plant most commonly used for all types of uses in Cuatrociénegas, Coahuila, Mexico

This lower species diversity is undoubtedly related to several factors, mainly the homogeneity of the landscape, consisting of a flat valley with a relatively homogeneous climate and soil with a high salt content [45], homogeneous vegetation, at least in the basin, as well as a much smaller area. Except for ornamental trees grown in the urban area, Cuatrociénegas practically lacks wild tree flora, except for several species of *Yucca*, *Prosopis*, and *Acacia*. Of the total species recorded, 95 are native and 61 are exotic. Ten main types of uses with their variants were registered, including ornamental (105 species), medicinal (98 species), food (52 species), forage (34 species), and construction (20 species); the remaining uses are given in Fig. 3. The parts most used by the local residents of Cuatrociénegas for different purposes are leaves, stems, fruits, inflorescences, and flowers; the remaining uses are given in Fig. 4 (Table 3; Supplementary Material).

#### Multifunctionality of ethnobotanical diversity

The diversity of ethnobotanical taxa (native and introduced) is used efficiently, satisfying a number of harvesting categories [46]. Because the collection of native species in the reserve is controlled and, in some cases, prohibited, a high inclusion of introduced species was observed. Plants have a pattern of multifunctional use; for example, it is observed that different parts of plants (fruits, flowers, and inflorescences) have different modes of use. This ethnobotanical multifunctionality, the number of reported species (*n* = 158), the type of ecosystem (Chihuahuan Desert), the mestizo communities, and the study area under the different protection categories all provide elements to support biocultural diversity in a broad sense, not restricted to the spatial correlation of the cultural, biological, and linguistic components [47]. Rather, it is made locally by the diversity of species (in this case, ethnobotany) included in the different socio-ecological systems [19]. In addition, traditional mestizo rural landscapes house biocultural heritage and play an important role in biodiversity conservation [20]. Moreover, the persistence of these rural landscapes depends directly on their maintenance and management, and traditional uses by the local population [48]. The importance of extrapolating the concept of biocultural diversity in urban sites as an explanation of the nature–society relationship must also be taken into account [21]; in this case, the relationship between traditional and semi-urban rural societies.

#### Ornamental

The 105 ornamental species, 64 native and 41 exotics, had the highest number of mentions of use in Cuatrociénegas. These plants play an important role in beautification of the regional landscape, mainly along streets and in public and private properties; the role of these cultivated plants in emerging countries has been reported [49]; they are commonly used as germoplasm reservoirs [50] and are found in multipurpose gardens [51], including medicinal, aesthetic, and edible [52]. Most ornamental species recorded are shrubs (39 species) and trees (37 species), while herbaceous plants account for 29 species. According to the interviewees, these elements were selected for one or several morphological, phenological, or phenotypic characteristics, highlighting the leaves (33 species), stems (28 species), inflorescences (20 species), or flowers (22 species), or a combination of several of these features (Supplementary Material); however, quantitatively, the tree species were the most commonly used and most frequently planted in many public squares and along sidewalks. *Fraxinus americana* was the most frequent species in streets, gardens and public squares, followed by *Morus celtidifolia*, especially the male plant since the female trees are not as preferred because when the fruits mature, they fall and stain the sidewalks when they are stepped on. Other common cultivated species are *Casuarina cunninghamiana*, *Platanus occidentalis*, *Carya illinoinensis*, *Eriobotrya japonica*, *Cupressus sempervirens*, and *Ligustrum japonicum*. In private gardens, the most frequent ornamental tree species were those that are also used as a source of food, for their edible fruits. Among these species are *Prunus persica*, *Persea americana*, *Prunus armeniaca*, *Punica granatum*, *Citrus sinensis*, *C*. *× limon*, *Populus alba*, and *Ficus carica*. Most of these species are also grown in southern Nuevo León, Mexico [40], and southern Mexico, including species of *Cucurbita* and *Citrus* as well as *Carica* and *Zea*. Some shrubby species are locally abundant in private gardens, notably *Nerium oleander* and *Casacabela thevetia.* Even empirically, people know about the toxicity of *Nerium olenader*, since it is known that two of its components, the cardiac glycosides olenadrine and neriine [53], could be deadly if ingested or even smoked. It is widely planted in many private gardens in Cuatrociénegas, and also in the south of Mexico. The toxic properties of *Cascabela thevetia* are also well known, being due to the cardenolides thevetin A and B [54]. There are no reported cases of poisoning caused by these species in the area. These two genera are used for the same purposes in Pakistan [55]*.* Accompanying these two species, the ornamental presence of several species and cultivars of *Rosa* is evident in gardens and along sidewalks.

#### Medicinal

Medicinal use was the second most important of the species reported in Cuatrociénegas, with 98 species, of which 39 are herbaceous, 43 shrubs, and 15 trees. There were 62 native and 36 introduced species. Similar percentages of growth forms, herbaceous, and shrub medicinal species were found in Ethiopia [56]. The families with the greater number of genera and species were Lamiaceae (10 genera and 11 species), Asteraceae (9 and 9), Cactaceae (8 and 15), and Lauraceae (3 and 4). Nine categories of use following the World Health Organization (WHO [57];) and 57 ailments or diseases treated were reported (Table 3). The main categories were digestive, integumentary, endocrine, respiratory, and circulatory (Supplementary Material).

Species used to alleviate digestive ailments stand out from the rest of the other uses, and almost 60% of the species are used exclusively for this purpose. The boiled leaves of 24 of these plants are used to alleviate ailments in a similar way to those reported in central Mexico [58], Bolivia [59], Ethiopia and Morocco [60, 61], and India [62]. Several native and exotic plants used in Mexico to alleviate digestive disorders such as *Tragia ramosa*, *Poliomintha glabrescens*, *Rosmarinus officinalis*, *Salvia officinalis*, *Peumus boldus*, and *Moringa oleifera* are used to alleviate these disorders around the world, for example in Turkey [63], Algeria [64], Serbia [65], and Nepal [66]. Among these medicinal species, several exotic species in Lamiaceae which have aromatic glands are notable, such as *Marrubium vulgare*, *Melissa officinalis*, *Mentha piperita*, *Mentha spicata*, *Ocimum basilicum*, as well as *Symphytum officinale*, *Citrus* × *limon*, *Citrus* × *sinensis*, and few autochtonous species such as *Artemisia ludoviciana*, *Poliomintha glabrescens*, *Persea americana*, and *Vachellia farnesiana*. Most of the exotic species are commonly used for medicinal purposes in southern Mexico [33], Colombia [67], and Europe (Serbia [68], Spain [69], and Bosnia-Herzegovina [70]). Almost half of the species used to alleviate digestive ailments (23) are exotic. The ancestral traditions about the use of these aromatic medicinal species for the cure of certain symptoms has been perpetuated by the pilgrimage of species, whose uses are repeated in different cultures and continents, as stated by Leonti and Casu [71]. This reinforces the hypothesis of transference between cultures, and ethnobotanical globalization and its ethnopharmacological knowledge.

The majority of dermal conditions or those related to the integumentary system are cured with at least 23 different species. Most of these species are native, mainly several genera such as *Cylindropuntia*, *Echinocactus*, *Echinocerus*, *Epithelantha*, *Ferocactus*, and *Opuntia*. These genera have a common use among the inhabitants of the area since the pulp of all of these plant species is used as a poultice to heal external wounds. These genera are of New World origin, but their traditional use is also found in other cultures where these species are introduced, such as in India [72]. These and other cactus genera are used for the same purposes in other countries. Some of these genera and species include *Opuntia* and *Melocactus* [73], *Opuntia* [74], and *Opuntia ficus*-*indica* [75]. Other important native species commonly used for these purposes are *Agave lechuguilla* (ground raw root); *Flourensia cernua* (boiled leaves), *Machaeranthera pinnatifida* (boiled leaves), and *Jatropha dioica* (raw root). *Agave lechuguilla* has a long tradition of use for the control of skin diseases [40]. It is known that the stems of *Jatropha dioica* are boiled and the resulting infusion is applied in the form of a poultice or used in baths to relieve infection from blows or external or internal wounds after washing with soap [41], and in Cuatrociénegas people use this plant in the same way. Similarly, the traditional medicinal uses of exotic species are the same or similar to those applied in their place of origin. Among these species noted for their widespread and multipurpose use to cure wounds are *Matricaria chamomilla*, *Aloe vera*, and *Punica granatum* [76, 77]. The treatments essentially involve the application of poultices with the solution obtained from the boiled, crushed or fresh plant parts.

The third place in importance as ranked by the number of mentions for medicinal uses is the use of plants to alleviate ailments of the endocrine system. There were 19 species in this category, 12 native and 7 exotics. The most common uses are the stems of the five *Opuntia* species, in addition to *Arctostaphylos pungens*, *Anemopsis californica*, *Solanum rostratum, Capsicum annum*, *Turnera diffusa*, *Lippia graveolens*, *Larrea tridentata*, and *Urtica chamedryoides*. Branches (14 species) and leaves (11 species), both boiled, and inflorescences (6 species) are the main plant parts used. These uses and species also occur in Bolivia [78].

Respiratory diseases are mainly treated with 13 species, half of which are native. Regardless of the part of the plant used, all these treatments involve the use of the boiled plant part and are taken as an infusion. The leaves are among the main plant parts used (*Rosmarinus*, *Eucaliptus*, *Citrus*, *Leucophyllum*, and *Poliomintha*), as well as stems (*Opuntia*) and bracts (*Bougainvillea*). It is often found that essential oils of *Citrus* are used to control cough [79], and the leaf tea and lemon juice of several genera of Rutaceae are good for eliminating cough [80]. *Eucalyptus* leaf tea is used in several countries to control respiratory ailments [81], commonly used in Cuatrociénegas.

Circulatory ailments are essentially treated with seven plants, five natives (*Ibervillea sonorae*, *Croton suaveolens*, *Portulaca oleracea*, *Leucophyllum frutescens*, and *Turnera diffusa*) and two exotic species (*Olea europea* and *Salvia officinalis*). The leaves, stems, and roots are the plant parts most used for these effects. The leaves and stems of these last two exotic species are boiled and drunk as an infusion. The dried root of *Ibervillea sonorae* (brought from the state of Sonora by plant sellers), cut into pieces and then added to water for later consumption, or the leaves, stems, and flowers of *Portulaca oleracea*, *Croton suaveolens*, and *Turnera diffusa* are commonly boiled and the infusion is drunk. These latter species are widely used in other areas of northeastern Mexico to purify the blood and increase physical strength [40–42]. *Croton* species have active alkaloids [82], and some species even produce red latex, which is culturally associated with certain medicinal properties [83]. The pink tones that the boiled water acquires when the branches of some species are added are considered an indicator that these plants are medicinal and they are frequently taken daily at lunchtime. This is the case for *Croton suaveolens*; when pieces of branches are added to boiling water, it acquires a pink hue, and is used as hot or iced tea as a daily drink instead of soft drinks as a way to purify the blood. Popular knowledge recognizes aphrodisiac properties of *Turnera diffusa* [41, 84]. Some interviewees mentioned that they use it daily to obtain better physical performance at work in the fields. Both virtues of this plant have been detailed in studies where at least twenty different chemical compounds have been detected [84]; however, it is still unknown which compound is responsible for the aphrodisiac activity [85], although the aphrodisiac effect has been demonstrated in rats [86]. Moreover, cultural affiliation diseases are also present in the mestizo communities and have been reported in other communities in northeastern Mexico [36–38]. The local people consider “the fright” as a health problem, for which branches with *Schinus molle* are used, passing through the whole body to clean and thus heal the sick. However, this health-disease connotation is different from that reported in indigenous communities [39]. We assume that it may be related to greater access to public and private health systems, so it is recommended to deepen its study in future works.

#### Food

The food category was the third most important group of plant species, accounting for 54 species. The plant parts used were fleshy and dry fruits (32), leaves (10), and seeds (9). There were 24 native species and 30 exotic species. Over half (51%) of the species were herbaceous and the rest shrubs or trees. The most commonly used were three natives (*Carya*, *Juglans*, *Persea*) and seven exotic species (*Ficus*, *Punica*, *Ziziphus*, *Cydonia*, *Eriobotrya*, *Prunus*, and *Citrus*). All these species are used with a dual purpose; on the one hand as a shade of fruit while the fruits are edible, or cooked to make sweets or syrups. At least seven of these double function genera are used in the same way in Morocco [87], and six of them as edible fruits [88]. The fruits of cultivated plants grown in the gardens are mainly for self-consumption, and sometimes fruits of *Ficus* and *Prunus* are sold at local markets. The fruits of native plants that are used the most are from the genera *Opuntia* and *Echinocereus*, which are picked in season to be consumed directly after removing thorns and husks or stored for a few days under refrigeration and later sold as seasonal fruit. Their sweet-sour pulp is used to make flavored ice pops or milk pops. There is a high demand for their seasonal consumption because they are products of a single season. Edible fruits of various genera of cacti such as *Opuntia*, *Hylocereus*, and *Stenocereus* are notable in the State of Mexico for being used much more than other families of native plants [89]. Our informants reported having a greater preference for these genera due to their presence most of the year or because they can store them dehydrated. This is partly consistent with the fact that people choose products that provide security, selecting species (products) present throughout the year [90], in addition to being a response to the availability of ethnobotanical resources present in the Cuatrociénegas region.

#### Forage

There were 21 forage species recorded; two exotic species, *Avena sativa* and *Sorghum bicolor*, which were the most important cultivated species used to feed domestic livestock, and 19 native wild species. The fruits and stems of all members of Cactaceae, and inflorescences and fruits of Asparagaceae (*Yucca*, *Dasylirion*, and *Agave*) are the most important wild species for forage. Except for *O*. *ficus-indica*, all the *Opuntia* fleshy stems are seared before feeding them to cattle, and the inflorescences, flowers, and edible fruits of all Aspraragaceae are eaten raw. *Opuntia ficus*-*indica* is common in the area and grown in many gardens. This species was domesticated in Mexico [91], and grows in human-modified environments [92]. It is common to find it in abandoned farming areas close to human settlements. It is used as fodder by cutting the stems with a machete to feed the cattle.

#### Construction and fuel

Twelve of the most common native genera in the regional landscape, including *Prosopis*, *Juniperus*, *Vachellia*, *Quercus*, *Cupressus*, *Fouquieria*, *Pinus*, *Fraxinus*, and *Larrea*, are the most common plants used for fuel and construction. They are used to build pens for cattle, attic, roofs, and columns for rural houses. Several of these genera are used similarly in southern Mexico [93] and North Africa [94]. Although the use of these woods is frequent, the areas bordering the natural protected area are not threatened, as the main source of fuel in the area is natural gas, sold in cylinders; however, from the point of view of economic botany, mesquite wood stands out as being economically important as it is sold for firewood and for the manufacture of handicrafts. It provides part of the income of a good number of inhabitants. Wooden crafts are sold mainly to domestic and foreign tourists. The prices of these items range from 30 pesos (about $1.60 USD) to 500 pesos (about $25.00 USD), according to the quality of the woodworking. The wood of the other species, *Vachellia*, *Quercus*, *Cupressus*, and *Pinus*, is regularly stored dry for cooking at gatherings of family and friends and it is used to roast goat, lamb, or pork.

#### Condiments

The native and exotic cultivated species used as condiments are an important part of traditional foods in Cuatrociénegas. In total, 14 species with seasoning properties were registered, 10 of them exotic, and four natives. Two plant families, Apiaceae and Lamiaceae, contain almost 77% of these species. Of the exotic species, the most notable for their multiple uses are *Mentha piperita*, *M. spicata*, *Coriandrum sativum*, *Petroselinum crispum*, *Ocimum basilicum*, *Rosmarinus officinalis*, and *Cuminum cyminum*. All of them are used daily in the preparation of various traditional dishes. Several of these species as well as many other different genera and species of this family are also used as medicinal plants to heal digestive disorders [95], or even to alleviate types of ailments such as respiratory and endocrine diseases [96]. Two of the most common native species used as condiments in the study area are *Capsicum annum* (to prepare spicy food) and *Lippia graveolens.* Both are also commonly present in many regional dishes; the latter is frequently used to prepare a heavily spiced dish (called menudo) considered to relieve hangovers. Although it is mentioned in few interviews, a boiled solution of the *Capsicum annuum* fruit is used as an anti-inflammatory. Some components of Capsaicinoides and capsinoids have anti-inflammatory activity [97]. An infusion of *Lippia graveolens* is often used in Cautrocienegas to alleviate phlegm produced by bacterial infections of the throat or sore throat, and it has been shown that this plant possesses antibacterial activity [98].

#### Living fences

A distinctive feature in rural homes in northern Mexico is the presence of live fences as a means of delimiting private property, especially small areas. Given the presence of a large number of shrub species with lateral or terminal spines, thorny fleshy stems, or hardwood, they are useful species for keeping cattle, native fauna, and humans away. These morphological characteristics are widely used for this purpose, and their attractive appearance, colorful and aromatic flowers, leaf size, and shape give them an additional aesthetic appeal, which also fulfills the function of beautifying an area as well as protecting it. The most common species used for these purposes are of the genera *Agave*, *Yucca*, *Fouquieria*, *Opuntia*, *Vachellia*, and *Prosopis*. *Agave* is the most effective due to its vegetative reproduction, producing young individuals adjacent to each other that make it very difficult for intruders to cross these fences.

#### Fibers

The production of fiber, which was once highly lucrative, today is a craft, which still survives; however, there are few people who are engaged in this activity, due to the low prices of crafts made from natural fiber. People say that it is not worth working so hard. This activity is less and less frequent in northern Mexico, even in the poorest communities [99]. The loss of this activity is a reflection of the transculturization processes.

### Use value of ethnobotanical species in Cuatrociénegas

#### Informant consensus factor (Fic), fidelity level (FL), and IVU (use value index)

Four species; three autochthonous, *Lophophora williamsii*, *Aricoarapus fissuratus*, *Cylindrountia imbricata*, and one exotic, *Sansevieria thyrsiflora*, obtained the highest FIC value (0.66). These species represent the osseous-muscular category; there is a high consensus in the use of these plants for the cure of this type of disease. *Lophophora* cut into pieces and dipped in alcohol has long been used as medicine in northeastern Mexico [41] and southern USA [100]. The other two categories of use with the highest FIC were the circulatory (0.5) and integumentary (0.5) categories (Table 4). Together, these two categories include 21 species (18 native). This points to the extensive empirical knowledge of the local people that results in continuous use of these species for the cure of certain related diseases; namely cacti to remedy integumentary ailments, and the species *Croton suavelones*, *Ibervillea sonorae*, *Portulaca oleracea*, and *Olaea europaea* to relieve circulatory ailments. Fourteen species with a high fidelity level were the most common species mentioned for healing a specific type of illness, mainly 11 native species, among them *Chenopodium ambrosioides* (antiparasitic, FL = 100%), *Poliomintha glabrescens* (cough, FL = 100%), *Jatropha dioica* (strengthening gums, FL = 85.7%), *Lophophora williamisii* (rheumatism, FL = 85.7%), and *Persea americana* (antiparasitic, FL = 83.3%). *Salvia officinalis* (anemia, FL = 75%) and *Mentha spicata* (stomach pain, FL = 60%) were the most mentioned exotic species (Table 5). The IVU values in this study range from 1.3 (*Matricaria chamomilla*) to 2.72 (*Larrea tridentata*). If it is considered that the IUV reflects the potential use of a particular plant to treat diseases, higher values of IVU indicate that the use of a particular species is more commonly used to alleviate particular illnesses. This is the case for the most important native species, such as *Larrea tridentata*, *Flourensia cernua* (IVU = 2.33), *Capsicum annuum* (IVU = 2.3), *Opuntia ficus*-*indica* (IVU = 2.25), *Opuntia engelmannii* (IVU = 2.23), *Turnera diffusa* (IVU = 2.13), *Hedeoma costata* (IVU = 1.9), and two exotic species, *Rosmarinus officinale* (IVU = 1.95) and *Mentha spicata* (IVU = 1.8), which, according to the information gathered, are considered the most reliable medicinal species in the cure of certain particular diseases (Table 6). Most interviewees who use *Larrea tridentata* as a medicine agree that it is useful for the removal of kidney stones and that the use of *Flouresnia cernua* is suitable for curing stomach pain. Most of the interviewees who know the medicinal use of *Capsicum annuum* apply it to eliminate cough. More than half of the interviewees mention that *Turnera diffusa* is an excellent remedy against body weakness. At least regionally in northeastern Mexico, these species are also used to alleviate the same or related illnesses [36, 40–42].

**Table 4:** Category of use, number of species mentioned (*nt*), number of uses recorded (*nur*), and FIC of medicinal plants used in Cuatrociénegas, Coahuila, Mexico. The Roman numerals correspond to the WHO International Statistical Classification of Diseases and Related Health Problems [57]

Category of use (system)	Number of species mentioned (*nt*)	*nur*	*Fic*
Digestive (XI)	48	68	0.29
Endocrine (IV)	18	29	0.39
Respiratory (X)	13	13	0.20
Integumentary (XII)	23	47	0.5
Circulatory (IX)	6	11	0.5
Nervous (VI)	1	2	0.01
Osseous-muscular (XIII)	2	4	0.66
Ocular (VII)	1	2	0.01
Reproductive (XIV)	1	5	0.01

**Table 5:** Fidelity label (*FL*) values obtained for the main medicinal plant species mentioned by informants in Cuatrociénegas, Coahuila, Mexico. *Ip* = number of informants who indicated the use of a plant for the same particular ilness; *Iu* = number of informants who mentioned the species for any illness within a category of use

Plant species	Ailment	*Ip*	*Iu*	*FL*
*Matricaria chamomilla* L., E (25066)	Stomach pain	26	45	57.7
*Machaeranthera pinnatifida* (Hook.) Shinners, N (25065)	Stomach pain	10	46	21.7
*Opuntia ficus-indica* (L.) Mill., N (25070)	Diabetes	7	13	53.8
*Turnera diffusa* Willd. ex Schult., N (25076)	Physical strength	6	11	54.5
*Litsea pringlei* Bartlett, N (24995)	Stomach pain	4	5	80
*Lophophora williamsii* (Lam. ex Salm-Dyck) J.M. Coult., N (25030)	Rheumatism	6	7	85.7
*Mentha spicata* L., E (25022)	Stomach pain	9	15	60
*Salvia officinalis* L., E (25020)	Anemia	3	4	75
*Jatropha dioica* Sessé, N (25059)	Strengthening gums	12	14	85.7
*Poliomintha glabrescens* A. Gray ex Hemsl., N (25053)	Cough	3	3	100
*Opuntia engelmannii* Salm-Dyck ex Engelm., N (25003)	Diabetes	18	27	66.6
*Persea americana* Mill., N (24978)	Antiparasitic	5	6	83.3
*Bougainvillea glabra* Choisy, N (25105)	Cough	26	36	72.2

**Table 6:** Medicinal plant species with the greatest number of different uses in Cuatrociénegas, Coahuila, Mexico and their respective IVU

Species (number of uses)	IVU	Species (number of uses)	IVU
*Larrea tridentata* (Sessé & Moc. ex DC.) Coville, N (6; 24999)	2.72	*Flourensia cernua* DC., N (3; 25035))	2.33
*Jatropha dioica* Sessé, N (4; 25059)	1.88	*Hedeoma costata* Hemsl., N (3; 25104))	1.9
*Machaeranthera pinnatifida* (Hook.) Shinners, N (4; 25065)	1.68	*Mentha spicata* L., E (3; 25022)	1.31
*Rosmarinus officinalis* L., E (4; 25021)	1.95	*Ruta graveolens* L., E (3; 24983)	1.44
*Artemisia ludoviciana* Nutt., N (3; 24097)	1.40	*Turnera diffusa* Willd. ex Schult., N (3; 25076)	2.13
*Opuntia engelmannii* Salm-Dyck ex Engelm., N (3; 25003)	2.23	*Aloe vera* L., E (3; 25041)	1.58
*Opuntia ficus-indica* (L.) Mill., N (3; 25070)	2.25	*Capsicum annuum* L., N (3; 25043)	2.3

## Conclusion

The native diversity of wild flora in Cuatrociénegas Valley, combined with the varied introduced flora, is an important multifunctional resource. Despite the fact that the local population is mestizo, these people have developed a complex inventory of knowledge of this flora and ethnobotanical practices adapted at a socio-ecological level. On the one hand, these practices address extreme desert environmental conditions, and secondly address socio-cultural processes of semi-urbanization. The ethnobotanical diversity yields a source of food, medicine, ornaments, timber, and other materials that are used depending on the needs of the local people. It is interesting to note the importance and care given to ornamental species, since, in other studies carried out elsewhere in semi-arid areas of northeastern Mexico [36, 40–42], it has not been reported. This special use of introduced ornamentals is, on the one hand, related to medicinal uses, but it is also especially due to the hostile desert ecosystem where the arid vegetation is mostly shrubs and herbaceous plants. For this reason, the local population strives to maintain gardens that provide them with shading for their grounds and houses, which allows them to mitigate, in part, the strong solar radiation and counteract the dry landscape. Another peculiarity was that although people give great importance to introduced species, the cultural importance index put native species characteristic of the Chihuahuan Desert in first place. This allows us to visualize the importance of the social-ecological role of the people in insuring the permanence of wild species. The diversity of native and introduced species, their multifunctionality, mestizo culture, semi-urbanization processes, desert ecosystem, as well as the conservation status and the flora collection restrictions at the Biosphere Reserve level make up complex ethnobotanical biocultural diversity of Cuatrociénegas. This constitutes an example supporting the concept that biocultural diversity is not only importane in regions with high biological and cultural diversity [44]. In an orthodox way, it has been mentioned that indigenous communities establish greater socio-ecological relationships; however, studies with mestizo communities, and the statistical comparison between them, determine that biocultural diversity is a much more complex system. Our results affirm that despite the peculiarities of Cuatrociénegas, native species are known and used. In addition, we show that there are cultural niches that are occupied and enhanced by a varied exotic flora. Therefore, biocultural diversity at the local level is a deep socio-ecological relationship, determined by multiple variables [19]. These variables describe the way people live and interact with nature, either in indigenous areas, in traditional rural landscapes [20], and even in urban landscapes [21], in this case, in semi-urban desert landscapes.

## Supplementary Information

**Additional file 1: Supplementary Material.** Plant families, genera, species and their uses in Cuatrocienegas, Coahuila, Mexico. The letter after author name indicates: N = Native, E = Exotic. The collection number belongs to the first author.

## Data Availability

Not applicable. Data sharing not applicable to this article as no datasets were generated or analused durin the current study.
