# Supplementary material for: Ethnobotanical biocultural diversity by rural communities in the Cuatrociénegas Valley, Coahuila; Mexico
Source: J Ethnobiol Ethnomed. 2021 Mar 29;17:21. doi: 10.1186/s13002-021-00445-0 (PMC8008621; doi:10.1186/s13002-021-00445-0)
Supplement: Supplementary file 1 — Additional file 1: Supplementary Material. Plant families, genera, species and their uses in Cuatrocienegas, Coahuila, Mexico. The letter after author name indicates: N = Native, E = Exotic. The collection number belongs to the first author. [file 13002_2021_445_MOESM1_ESM.pdf]

Estrada E. et al. 2021. Ethnobotanical biocultural diversity by rural communities in the Cuatrociénegas Valley, Coahuila; Mexico. Journal of Ethnobiology and Ethnomedicine.

Supplementary Material. Families, genera, species and their uses in Cuatrocienegas, Coahuila, Mexico. The letter after author name indicates: N = Native, E = Exotic. The collection number belongs to the first author.

Scientific name	Common name	Uses (system)	Part used	Method of use
**ACANTHACEAE**				
Beloperone gutatta Brendegee, N, 24957	Camarón	Ornamental	Whole plant for the beauty of its flowers	Planted in private and public gardens
**ADOXACEAE**				
Sambucus nigra L., N, 24988		Ornamental	Whole plant for their showy inflorescences and fragrant flowers	Planted in private and public gardens
		Medicinal (respiratory system), cough	Inflorescens and flowers	Boiled, drink as infusion
**ALLIACEAE**				
Allium cepa L., E, 24956	Cebolla	Medicinal (digestive, system), colic,	Stems	Boiled, 1apónica1 of infusion
		Medicinal (respiratory), runny nose	Stems	Boiled, 1apónica1 of infusion
Allium sativum L., E, 25039	Ajo	Condiment	Stems (cloves)	Macerated, raw or boiled
**AMARANTHACEAE**				
Amaranthus blitoides S. Watson, N, 24968	Quelite	Food	Leaves and tender stems	Boiled, cook with eggs
Atriplex canescens (Pursh) Nutt., N, 25040	Chamizo, costilla de vaca	Forage	Whole plant	Raw
Celosia crista L., N, 25000	Mano de león	Ornamental	Whole plant, and by its showy inflorescences	Planted in private gardens
		Medicinal (digestive system), stomach ache	Leaves and inflorescences	Boiled, drink the infusion
Dysphania ambrosioides (L.) Mosyakin & Clemants, N, 24969		Food	Leaves	Flavor foods

		Medicinal (Digestive system), antiparasitic	Leaves	Boiled, 2apónica2 of 2apónica
Spinacia oleracea L., E, 25060	Espinaca	Food	Leaves	Cook or boiled
**ANACARDIACEAE**				
Schinus molle L., E, 24955	Pirúl	Ornamental	Whole plant, selected by its perennial foliage	Planted in private and public gardens
		Religious rites, fright	Leaves	Shaking and lightly touching the body with the leaves while praying
**APIACEAE**				
Coriandrum sativum L., E, 24958	Cilantro	Condiment	Leaves	Boiled or raw
		Medicinal (digestive system), stomach ache	Leaves	Boiled, drink the infusion
Cuminum cyminum L., E, 25061	Comino	Condiment	Seeds	Boiled with food
Daucus carota L., E	Zanahoria	Food	Roots	Raw or boiled
Petroselinum crispum (Mill.) Fuss, E, 25062	Perejil	Condiment	Leaves	Boiled or raw
**APOCYNACEAE**				
Nerium oleander L.,E, 25100	Laurel	Ornamental	Whole plant, perennial folage and showy flowers	Planted in private and public gardens
Cascabela thevetia (L.) Lippold, E, 24954		Ornamental	Whole plant	Planted in public and private gardens
Vinca minor L., E, 25099	Teresita	Ornamental	Whole plant, and beautiful flowers	Planted in private and public gardens
**ARECACEAE**				
Washingtonia robusta H. Wendl., N, 25001	Palma	Ornamental	Whole plant, by its perennial big leaves	Planted in private and public gardens
**ASPARAGACEAE**				
Agave lechuguilla Torr., N, 25038	Lechuguilla, amole	Medicinal (Integumentary system), external wounds	Root	Milled (pulp) and fresh, as soap or shampoo, as poultice on the affected part
		Craft	Fibers of the	Dried, woven

			leaves	to make sacks
Agave parrasana A. Berger, N, 25098	Maguey	Ornamental	Whole plant, beautiful leaves	Planted in private and public gardens
		Food	Sap	Raw or cook, drink to accompany meals
		Liquor	Sap	Root, fermented
		Condiment	Leaves	The meat wrapped in maguey leaves is placed in the oven under the ground and left for a full night, barbecue type
Agave scabra Ortega, N, 25063	Maguey	Ornamental	Whole plant, beautiful leaves	Planted in private and public gardens
		Medicinal (respiratory system), pneumonie	Leaves (pulp)	Boiled, drink as tea
		Food	Sap	Raw or cook, drink to accompany meals
Dasylirion cedrosanum Trel., N, 25002	Sotol	Ornamental	Whole plant, beautiful leaves	Planted in private and public gardens
		Liquor	Root	Fermented
		Fibers	Leaves	Dried, moistened and separated in thin fibers to crafts
Sansevieria thyrsiflora (Petagna) Thunb., E, 24970	Lengua de suegra, guaco	Ornamental	Whole plant, perennial and showy inflorescence	Planted in private properties and public gardens
		Medicinal (integumentary system), dermical wounds	Leaves	Milled (pulp) and fresh, poultice on the affected part
		Medicinal (Muscular system), muscle pain	Leaves and roots (pulp)	Raw, apply as a poultice
Yucca torreyi Shafer, N, 25037	Palma	Ornamental	Whole plant, its beautiful stem and showy flowers	Planted in private properties and public gardens
		Food	Fruits	Raw

Yucca trecuelana Carriere, N, 24971	Palma	Ornamental	Whole plant, beuatiful flowers and leaves	Planted in private properties and public gardens
	Palma	Food	Flowers	Boiled or cook, mixed with eggs, pepper and onion
		Food	Fruits	Raw
**ASTERACEAE**				
Ageratina havanensis (Kunth) R.M. King & H. Rob., N, 25036	Ageratina	Ornamental	Whole plant, beautiful inflorescences	Planted in private properties
Artemisia ludoviciana Nutt., N, 25097	Estafiate	Medicinal (digestive system), colic, stomach ache	Branches, inflorecences, flowers	Boiled, 4apónica4 of infusion
		Medicinal (digestive system), indigestion	Leaves	Chew and swallow raw leaves
		Medicinal (digestive system), diarrhea	Levaes	Boiled, drink the infusion
Calendula officinalis L., E, 24972	Cartulina	Ornamental	Whole plant, showy heads	Planted in private properties and public parks
		Medicinal (respiatory system), cough infections	Leaves and inflorescences	Boiled, drink the infusion
Chrysactinia mexicana A. Gray, N, 24987	Hierba de San Nicolás	Medicinal (reproductive system), sexual strenght	Leaves	Boiled, drink the 4apónica (this plant is frequently mistaken with *Turnera diffusa*)
Cynara scolymus L., E, 24959	Alcachofa	Medicinal (Circulatory system), reduce cholesterol	Leaves	Milled
		Medicinal (endocrin system), cleanse the liver	Young leaves and flowers	Boiled, drink the infusion
Flourensia cernua DC., N. 25035	Hojasé	Medicinal (integumentary system), bad smell of feet	Dry leaves	Put some leaves inside the shoes
		Medicinal (digestive system), laxative	Leaves	Boiled, 4-5 leaves, infusión, strong drink

		Medicinal (digestive system), stomach pain	Hojas	Boiled, 4-5, small sip, strong drink
Gnaphalium semiamplexicaule DC., N, 25064	Gordolobo	Medicinal (respiratory system), cough	Inflorescences and flowers	Boiled, drink the infusion
Lactuca sativa L., E, 24960	Lechuga	Food	Leaves	Raw, to prepare salads
Machaeranthera pinnatifida (Hook.) Shinners, N, 25065	Árnica	Medicinal (integumentary system), external wounds	Leaves and inflorescences	Milled, as poultice on the affected part
		Medicinal (digestive system) ulcers	Whole plant	Boil, drink the infusion
		Medicinal (integumentary system) disinfect and heal wounds	Whole plant	Boils, solution used as poultice
		Medicinal (integumentary system), pimples	Branches	Apply the solution directly in the affected area
Matricaria chamomilla L., E, 25066	Manzanilla	Medicinal (digestive system), stomach ache, colics	Leaves and flowers	Boiled, drink the infusion
		Medicinal (ocular system), clean eyes	Leaves and flowers	Put two warm drops of the 5apónica directly in the eye
Parthenium argentatum A. Gray, N, 25095	Guayule	Ornamental	Whole plant, color and shape of leaves	Planted in private properties and public parks
Parthenium incanum Kunth, N, 25094	Guayule	Ornamental	Whole plant, color of leaves	Planted in private properties and public parks
		Medicinal (digestive system), stomach pain	Whole plant	Milled, boiled, drink the 5apónica, a small sip, strong flavor
Tagetes erecta L., N, 24973	Cempazuchitl	Ornamental	Whole plant, showy orange to yellow heads	Planted commonly in public parks
**BIGNONIACEAE**				
Chilopsis linearis (Cav.) Sweet, N, 24986	Mimbre	Ornamental	Whole plant, big growth and showy purple flowers	Planted in private properties and public parks,

				by its beautiful flowers
Tecoma stans (L.) Juss. ex Kunth, N, 25034	San Pedro	Ornamental	Whole plant, showy yellow flowers	Commonly planted in private gardens, by its beautiful flowers
**BORAGINACEAE**				
Symphytum officinale L., E, 24974	Suelda	Ornamental	Whole plant, showy flowers	Commonly planted in private gardens, by its beautiful flowers
		Medicinal (digestive system), diarrhea	Root	Boiled, drikn as tea
**BRASSICACEAE**				
Beta vulgaris L., E, 24961	Betabel	Food	Root	Raw or boiled, cut into pieces
Raphanus sativus L, E., 25033	Rábano	Food and condiment	Root	Raw, cut into pieces
**CACTACEAE**				
Ariocarpus fissuratus (Engelm.) K. Schum., N, 25091	Chaute	Ornamental	Whole plant, perennial, perennial stems	Planted in private gardens
		Medicinal (Muscular system), pains	Stems	Raw pulp, as poultice
Coryphantha pseudoechinus Boed., N, 25092	Chilitos	Ornamental	Whole plant, its cylindric shape, perennial	Planted in private gardens
Cylindropuntia leptocaulis (DC.) F.M. Knuth, N, 24985	Tasajillo	Ornamental	Whole plant, its long perennial stems	Planted in private gardens
		Medicinal (Muscular system)	Stems	Raw, pulp, as poultice
Cylindropuntia imbricata (Haw.) F.M. Knuth, N	Cardenche	Ornamental	Whole plant, its long stems	Planted in private gardens
Echinocactus horizonthalonius Lem. , N, 25093	Manca caballo	Ornamental	Whole plant, its sphaerical shape	Planted in private gardens
		Medicinal (integumetary system), wounds	Pulp of stems	Raw
Echinocactus platyacanthus Link & Otto, N, 24989	Biznaga burra	Ornamental	Whole plant, its big size and sphaerical shape	Planted in private gardens

		Forage	Stems	Raw, cut into pieces
		Food	Stems	Boiled with sugar, carmalized desserts
		Medicinal (integumentary system), external wounds	Pulp of stems	Raw
Echinocactus texensis Hoppfer, N, 25032	Manca caballo	Ornamental	Whole plant, its sphaerical shape	Planted in private gardens
		Medicinal (integumentary system), external wounds	Pulp of stems	Raw
Echinocereus enneacanthus Engelm., N, 25090	Pithaya	Food	Fruits	Raw, remove the peel and eat
		Food	Fruits	Mixed with milk to make ice cream
		Medicinal (integumentary system), external wounds	Pulp of stems	Raw
		Forage	Stems	Raw
Echinocereus pectinatus (Scheidw.) Engelm., N, 25051	Pithaya	Ornamental	Whole plant, by its cylindrical, and abundant stems	Planted in private gardens
		Medicinal (integumentary system), external wounds	Pulp of stems	Raw
		Forage	Stems	Raw
Epithelantha micromeris (Engelm.) Weber, N, 25067	Biznaguita blanca	Ornamental	Whole plant, by the color of its stems, almost thornless	Planted in private gardens
		Medicinal (integumentary system), wounds	Pulp of stmes	Raw
Ferocactus pilosus (Engelm.) F.A.C. Weber ex Britton & Rose, N, 24990	Barril de fuego	Ornamental	Whole plant, by its evident and conspicuous red color prickles	Planted in private gardens
		Forage	Stems	Raw, cut into pieces
		Food	Dried fruits called cabuches	Raw or boiled and stored in brine, eat

				directly
		Medicinal (integumentary system), external wounds	Pulp of stems	Raw
Lophophora williamsii (Lam. ex Salm-Dyck) J.M. Coult., N, 25030	Peyote	Medicinal (osseous and muscular systems), rheumatism	Whole plant into alcohol mixed with *Cannabis indica*, *Rosmarinus officinalis* and *Ruta graveolens*	As poultice in the affected area
		Ornamental	Whole plant, by its flattened shape	Planted in private gardens
Opuntia engelmannii Salm-Dyck ex Engelm., N, 25003	Nopal	Forage	Stems	Raw, scorched to remove thorns
		Food	Fruits	Raw
		Food	Fruits	Boiled, to prepare jam.
		Medicinal (endocrin system), diabetes control	Stems	Raw (pulp), cook with olive oil
		Medicinal (integumentary system), wounds and burns	Stems (pulp)	Raw, directly on the affected or wounded area
		Medicinal (digestive system), stomach ache	Pulp	Raw
Opuntia ficus-indica (L.) Mill., N, 25070	Nopal criollo	Forage	Stems	Raw
		Medicinal (endocrin system), diabetes control	Stems	Raw (pulp), cook with olive oil
		Ornamental	Whole plant, by its long grwoth and thornless	Palnted in private gardens
		Medicinal (digestive system), stomach ache	Pulp	Raw
		Medicinal (respiratory system), respiratory diseases	Root	Milled and boiled, drink the infusion
Opuntia grahamii Engelm., N, 25004	Nopal	Forage	Stems	Raw, scorched to remove

				thorns
		Medicinal (digestive system), stomach ache	Pulp	Raw
		Medicinal (endocrin system), diabetes control	Stems	Raw (pulp),
Opuntia imbricata (Haw.) DC., N. 25029	Coyonoxtle	Medicinal (digestive system), stomach ache	Fruits	Boiled, drink the pulp
		Medicinal (endocrin system), diabetes control	Stems	Raw (pulp),
Opuntia phaeacantha Engelm., N, 25028	Nopal rastrero	Forage	Stems	Raw, scorched to remove thorns
		Medicinal (digestive system), stomach ache	Pulp	Raw
		Medicinal (endocrin system), diabetes control	Stems	Raw (pulp), or cook with olive oil
**CANNABACEAE**				
Celtis pallida Torr., N, 25089	Granjeno	Food	Fruits	Raw
		Food	Fruits	Boiled, to make jam
**CAPRIFOLIACEAE**				
Lonicera japonica Thunb., N, 25005	Madreselva	Ornamental	Whole plant, by its showy and the rich aroma of its flowers	Planted in private properties
**CARCIACEAE**				
Carica papaya L., 24962	Papaya	Ornamental	Whole plant	Planted in private gardens
		Food	Fruit	Raw
		Medicinal (Disgestive system), antiparasitic	Seed	Raw, milled, drink
**CASUARINACEAE**				
Casuarina cunninghamiana Mig., E, 25027	Casuarina	Ornamental	Whole plant, by its large growth and perennial foliage	Planted in public parks
		Construction	Trunks and branches	Dry wood

**CUCURBITACEAE**				
Cucurbita pepo L., N, 25068	Calabaza	Food	Fruit	Cook, caramelized snacks
			Seeds	Dried and salted, as snacks
Ibervillea sonorae (S. Watson) Greene, N, 24963	Wereke	Medicinal (circulatory system), colesterol reduce	Root	Dry, raw, cut into slices, eat two slices
Citrullus lanatus (Thunb.) Matsum. & Nakai, E	Sandía	Food	Fruit	Raw, cut into slices
Cucumis melo L., E, 25026	Melón	Food	Fruit	Raw, cut into slices
			Seeds	Dried, to prepare horchata water
Cupressus arizonica Greene, N, 25006	Ciprés	Ornamental	Whole plant, its large size and pereennial foliage	Planted in public parks and private gardens
		Fuel	Trunks and branches	Dry
Cupressus sempervirens L., E, 25007	Pincel	Ornamental	Whole plant, its large size and pereennial foliage	Planted in public parks
		Fuel	Trunks and branches	Dry
Juniperus flaccida Schltdl., N, 25008	Táscate	Ornamental	Whole plant, its large size and pereennial foliage	Planted in public parks
		Fuel	Trunks and branches	Dry
**ERICACEAE**				
Arctostaphylos pungens Kunth, N, 25101	Pingüica	Medicinal (endocrin system), kidney diseases	Leaves	Boiled, ingestion of infusion
**EUPHORBIACEAE**				
Cnidoscolus aconitifulius (Mill.) I.M. Johnst., 25025	Chaya	Ornamental	Whole plant (leaves)	Planted in private gardens
		Medicinal (digestive system)	Leaves	Raw, milled mixed with water, juice
Croton suaveolens Torr., N, 25087	Salvia	Medicinal (circulatory system), improve blood circulation	Leaves and branches	Boiled, use as a common drink
Euphorbia antisyphilitica Zucc., N, 25088	Candelilla	Industrial (sap)	Sap	Boiled and dried, to obtain crude wax

Jatropha dioica Sessé, N, 25059	Sangre de drago, sangre de grado	Medicinal (digestive system), to prevent teeth from falling, strengthening gums	Root	Biting the root (do not swallow the sucked sap every single morning) for 15 days
		Medicinal (ocular system), clean eyes	Stems	Apply a drop of sap to the eyes
		Medicinal (integumentary system), avoid hair loss	Sap	As shampoo, apply directly on the hair
		Medicinal (Integumentary system) skin wound	Sap	As poultice in the affected area
Ricinus communis L., E, 25057	Higuerilla	Medicinal (digestive system), colic, stomach infections	Leaves	Boiled, ingestion of infusion
Tragia ramosa Torr., N, 25058	Mala mujer	Medicinal (digestive system), stomach ulcers	Leaves	Boiled, ingestion of infusion
		Medicinal (Endocrin system), vaginal infections	Whole plant	Boiled, drink the infusion
**FABACEAE**				
Acacia farnesiana (L.) Willd., N, 24991	Huizache	Forage	Pods and seeds	Raw
		Medicinal (digestive system), indigestion	Seeds	Toasted
		Construction	Wood	Dry, for roofs, fences and clumns for cabins
Caesalpinia mexicana A. Gray, N, 25102	Hierba del potro	Ornamental	Whole plant, by its showy inflorescences and flowers	Planted in private properties
Dalea bicolor Willd., N, 24992	Engorda cabras	Forage	Whole plant	Raw, mainly for goats
Eysenhardtia texana Scheele, N, 25056	Vara dulce	Forage	Leaves and branches	Raw, domestic livestock
Phaseolus vulgaris L., N	Frijol	Food	Seeds	Cook
Prosopis glandulosa Torr., N, 25103	Mezquite	Forage	Pods and seeds	Raw, for goats and horses mainly
		Construction	Wood	Dry, for roofs, fences and

				clumns for cabins
		Food	Seeds	Toasted and milled (flour), squeeze and make sweets in molds
		Handcrafts	Wood	Dry
Vicia faba L., E, 25024	Haba	Food	Seeds	Cook
**FAGACEAE**				
Quercus spp., N, 25055	Encino	Construction	Wood	Construction of country cottages
**FOUQUIERIACEAE**				
Fouquieria splendens Engelm., N. 25069	Albarda, ocotillo	Construction	Stems	Corrals for domestic livestock
**GERANIACEAE**				
Pelargonium zonale (L.) L'Hér. ex Aiton, E, 25023	Geranio	Ornamental	Whole plant, by the polichromatic flowers	Planted in many private gardens
**JUGLANDACEAE**				
Carya illinoinensis (Wangerin) K. Koch, N, 25054	Nogal	Food	Fruit	Raw
		Medicinal (integumentary system) hair dye	Fruit (husk)	Raw, put parts into water, wait for 12 hours and apply directly on the hair
		Food	Fruits	Caramelized walnuts
Juglans microcarpa Berl., N	Nogalillo	Construction	Wood	Manufacturing household goods
Juglans major (Torr.) Heller, N , 24993	Nogal de nuez encapsulada	Food	Fruit	Raw
**LAMIACEAE**				
Hedeoma costata Hemsl., N, 25104	Poleo	Condiment	Leaves	Cook
		Medicinal (nervous system), eliminate nervous tension	Leaves	Boil, drink as tea
		Medicinal (digestive system), cure stomach of any discomfort	Leaves	Boil, regrigerate, drink frost preparation
		Medicinal (digestive	Leaves	Boil and drink infusion

		system), constipation		
Majorana hortensis Moench, E, 24964	Mejorana	Condiment	Leaves	Mix with food
		Condiment	Leaves	To prepare pesto (garlic and olive oil) in restaurants
		Medicinal (digestive system), constipation	Leaves	Boil and drink infusion
Marrubium vulgare L., E, 25086	Marrubio	Medicinal (digestive system), stomacha ache	Leaves	Boil and drink infusion
Melissa officinalis L., E, 24975	Toronjil	Remove mosquitoes from home	Whole plant	Scorched dry leaves
		Medicinal (digestive system), stomacha ache	Leaves	Boil and drink infusion
Mentha x *piperita* L., E, 24976	Yerbabuena	Condiment	Leaves	Boil with beaf and soup
		Medicinal (digestive system), cure stomach of any discomfort		Boil and drink infusion
Mentha spicata L., E, 25022	Yerbabuena	Condiment	Leaves	
		Medicinal (digestive system), cure stomach of any discomfort		Boil and drink infusion
		Medicinal (nervous system), insomnia	Leaves and inflorescences	Boiled, drink the infusión by night
		Medicinal (digestive system), bad breath	Raw or boiled	Chew leaves or boiled, drink the infusión and gargle
Ocimum basilicum L., E, 24994	Albahaca	Condiment	Leaves and inflorescences	Boil and drink infusion
		Food	Leaves	Raw, in salads
		Medicinal (auditive system)	Leaves	Introduce a leave into the ear
		Medicinal (digestive system), cure stomach of any discomfort	Leaves and inflorescences	Boil and drink infusion
Poliomintha glabrescens A. Gray ex Hemsl., N, 25053	Orégano	Medicinal (digestive	Leaves	Boil and drink infusion

		system), constipation		
		Condiment	Leaves	Add to the “menudo” (mix of viscera in broth with spicy)
Rosmarinus officinalis L., E, 25021	Romero	Condiment	Leaves	Cook together with the barbecue
		Medicinal (digestive system), constipation	Leaves	Boil and drink infusion
		Medicinal (respiratory system), stuffy nose	Leaves and branches	Dried, milled, scorch
		Medicinal (endocrin system), pains during menstruation	Leaves and inflorescences	Mixed with *Cinnamomum verum* (canela)
Salvia officinalis L., E, 25020	Salvia real	Condiment	Leaves and inflorescences	Cook together with the food
		Medicinal (reproductive system), vaginal infections	Whole plant	Boiled, drink as tea
		Medicinal (Circulatory system), anemia	Leaves and branches	Take on an empty stomach, drink as tea for a month
Thymus vulgaris L., E, 24965	Tomillo	Condiment	Leaves and flowers	Add to the food when it is cooking
		Medicinal (digestive system), constipation	Leaves and stems	Dry, boil and drink infusion
**LAURACEAE**				
Cinnamomum verum J. Presl., E, 25009	Canela	Condiment	Bark	Add to oatmeal gruel together with honey bee
Litsea pringlei Bartlett, N, 24995	Laurel	Medicinal (digestive system), stomach pain	Leaves	Dry, but boiled, drink the infusion
		Medicinal (digestive system), inflamed stomach	Leaves	Boiled, drink the infusion
Persea americana Mill., N,	Aguacate	Food	Fruit	Raw, pulp

24978				
Persea americana Mill. var *drymifolia* (Schltdl. & Cham.) S. F. Blake, N, 24977	Aguacate criollo	Medicinal (digestive system), antiparasitic	Fruit peel	Raw
**LILIACEAE**				
Asparagus officinalis L., E, 25010	Aspárago	Ornamental	Whole plant	Planted in private gardens
**LYTHRACEAE**				
Punica granatum L., E, 24979	Granada	Medicinal (integumentary system), external wounds	Peel	Raw, rub the affected area
		Food	Fruit	Raw
		Food	Fruit	Beverage, juice, raw
		Food	Fruit	Liquor, fermented
		Food	Fruit	Boiled, to make jam
**MALVACEAE**				
Gossypium hirsutum L., N, 24996	Algodón	Ornamental	Whole plant, by its beautiful foliage and flower	Planted in private gardens
		Fibers	Fruit	Raw fibers, dried and open fruits
Hibiscus rosa-sinensis L., E, 24966	Hibisco	Ornamental	Whole plant, its beautiful leaves and showy flowers	Planted in private gardens
Hibiscus syriacus L., E, 25011	Rosa de Siria	Ornamental	Whole plant, its beautiful leaves and showy flowers	Planted in private gardens
**MELIACEAE**				
Melia azedarach L., 24967	Canelón lila	Ornamental	Whole plant	Planted in public parks and privtae gardens
		Fuel	Trunks and branches	Dry
**MONIMIACEAE**				
Peumus boldus, E, 25019	Boldo	Ornamental	Whole plant, by its flowers and leaves	Planted in private gardens
		Medicinal (disestive system), constipation, flatulences	Leaves	Boiled, ingestion of infusion

		Medicinal (endocrin system), detosxify liver	Dried leaves	Boiled, drink as tea
**MORACEAE**				
Ficus carica L., E, 25012	Higuera	Ornamental	Whole plant, by its perennial leaves	Planted in private gardens
		Food	Infrutescences (pseudo fruits)	Raw
Morus celtidifolia Kunth, N, 25085	Mora	Ornamental	Whole plant, by its abundant foilage	Planted in private gardens and public parks
		Food	Fruit	Raw
**MORINGACEAE**				
Moringa oleifera Lam., E, 25071	Moringa	Ornamental	Whole plant, by its flowers	Planted in private gardens
		Medicinal (digestive system), stomach ache	Leaves	Milled, boiled, ingestion of infusion
		Medicinal (endocrni system), diabetes	Leaves	Boiled, drink the infusion
**MYRTACEAE**				
Eucalyptus camaldulensis Dehnh.,E, 25018	Eucalipto	Medicinal (respiratory system), bronchitis	Leaves and fruits (dried)	Boiled, as tea mixed with lemon
		Fuel	Trunks and branches	Dry
Eucalyptus globulus Labill., E, 25072	Eucalipto	Medicinal (respiratory system), bronchitis	Leaves and fruits (dried)	Boiled, as tea, mixed with lemon
		Fuel	Trunks and branches	Dry
**NYCTAGINACEAE**				
Bougainvillea glabra Choisy, N, 25105	Bugambilia	Ornamental	Whole plants, by its showy and polichromatic bracts	Planted in many places, private and public ones
		Medicinal (digestive and respiratory systems), stomach ache and throat inflammation	Bracts	Booiled, ingestion of infusion

Mirablis jalapa L., N, 25052	Maravilla	Ornamental	Whole plant, by its beautiful yelow and large flowers	Cultivated in private gardens
**OLEACEAE**				
Fraxinus americana L, N, 25017	Fresno	Ornamental	Whole plant, by its large size and long leaves	Cultivated in private gardens and public parks
		Construction	Trunks	Dry
Ligustrum japonicum Thunb, E, 25051	Trueno	Ornamental	Whole plant, by its perennial leaves and dense inflorescences	Cultivated in private gardens and public parks
Olea europea L, E, 25106	Olivo	Ornamental	Whole plant, by its leaves	Cultivated in private gardens
**PINACEAE**				
Pinus cembroides Zucc., N, 25049	Pino piñonero	Ornamental	Whole plant, by its aroma, and perennial leaves	Cultivated in private gardens
		Construction	Trunks	Dry
		Food	Seeds	Raw
Pinus pinceana Gordon, N, 25050	Pino	Ornamental	Whole plant, by its aroma, and perennial leaves	Cultivated in private gardens
		Construction	Trunks	Dry
**PLATANACEAE**				
Platanus occidentalis L., N, 25107	Álamo	Ornamental	Whole plant, by its beautiful leaves	Planted in public parks
**POACEAE**				
Arundo donax L., E, 25108	Carrizo	Construction	Stems	Dry, household goods
Avena sativa L., E, 25109	Avena	Food	Seeds	Cook
		Medicinal (digestive system), colic	Seed	Oatmeal gruel
		Forage	Whole plant	Dry, for domestic livestock
Hordeum vulgare L., E, 25048	Cebada	Forage	Whole plant	Dry, for domestic livestock
Sorghum bicolor (L.) Moench, E	Sorgo	Forage	Whole plant	Dry, for domestic livestock
Zea mays L., N, 25047	Maíz	Food	Seeds	Cook, to make dough and then tortillas
		Forage	Whole plant	Dry, for

				domestic livestock
		Medicinal (digestive system), to eliminate worms	Styles	Boil, drink the infusion
		Medicinal (endocrin system) kidney diseases	Styles	Boil, drink the infusion
**PORTULACACEAE**				
Potrulaca oleracea L., N, 25073	Verdolaga	Medicinal (circulatory system), to improve blood circulation	Leaves	Raw or boiled, eat and drink the infusion
**PTERIDACEAE**				
Adiantum capillus-veneris L., N, 25110	Culantrillo	Ornamental	Whole plant, by its beautifl leaves	Planted in private gardens
Argyrochosma limitanea (Maxon) Windham, N, 25074	Helecho	Ornamental	Whole plant, by its beautifl leaves, and pubescence color	Planted in private gardens
Asplenium exiguum Bedd., N, 25046	Helecho	Ornamental	Whole plant, by its beautifl leaves	Planted in private gardens
Pleopeltis guttata (Maxon) E.G. Andrews & Windham, N	Helecho	Ornamental	Whole plant, by its beautifl leaves	Planted in private gardens
**RHAMNACEAE**				
Ziziphus jujuba Mill., E, 25084	Jujube	Ornamental	Whole plant, by its leaves	Plantd in private properties, rare
		Food	Fruit	Raw
**ROSACEAE**				
Cydonia oblonga Mill., E, 25083	Membrillo	Food	Fruit	Raw or boiled
		Ornamental	Whole plant, by its beautiful growth	Planted in private properties
		Medicinal (digestive system), colic	Fruit	Boild, drink the pulp
		Food	Fruit	preserved fruits (canned)
Eriobotrya japonica (Thunb.) Lindl., E, 24980	Níspero	Food	Fruit	Raw
		Ornamental	Whole plant, by tts large size and used as shadow tree	Planted in private properties
Prunus armeniaca L., E, 25045	Chabacano	Food	Fruit	Raw

		Ornamental	Whole plant, by its stem color and testure of its leaves	Planted in private properties
Prunus domestica L., E, 25082	Ciruelo	Food	Fruit	Raw
		Ornamental	Whole plant, by its leaves and fruits	Planted in private properties
Prunus persica (l.) Batsch, E, 25081	Durazno	Food	Fruit	Raw
		Ornamental	Whole plant, by its dense foliage	Planted in private properties
		Food	Fruit	Boiled to make jam
Rosa gallica L., E, 25014	Rosa	Ornamental	Whole plant, by its showy flowers	Planted in private properties
Rosa sp., E, 25013	Rosa	Ornamental	Whole plant, by its showy flowers	Planted in private properties
**RUTACEAE**				
Citrus limon (L.) Osbeck, E, 24982	Limón	Food, hydration	Fruit	Raw, limonade
		Ornamental	Whole plant, by its persistent foliage and dense foliage	Planted in private properties
		Medicinal (digestive and respiratory systems), stomach discomfort and cough	Leaves	Boiled, as tea
Citrus x *sinensis* (L.) Osbeck, E, 24981	Naranja	Food	Fruit	Raw
		Medicinal (digestive system), stomach discomfort	Leaves	Boiled, as tea
		Ornamental	Whole plant, by its persistent foliage and dense foliage	Planted in private properties
		Medicinal (respiratory system), cough	Leaves	Boiled, drink the infusion

Ruta graveolens L., E, 24983	Ruda	Medicinal (digestive system), colic	Leaves, in warm water for 3 minutes only	Boiled, drink infusion
		Condiment	Leaves	Milled, added to the food
		Medicinal (endocrin system)	Whole plant	Miled, boiled, drink the infusion
		Medicinal (digestive system), stomach ache	Branches	Boiled, drink the infusion
**SALICACEAE**				
Populus alba L., N, 25015	Álamo	Ornamental	Whole plant, by the color of its levaes (white adaxially)	Planted in private properties and public areas
Salix nigra Marshall, N,2 5016	Sauce	Ornamental	Whole plant, by its narrow leaves	Planted in private properties and public parks
**SAPINDACEAE**				
Aesculus hippocastanum L., E, 25044	Castaño de la Indias	Medicinal (circulatory system) blood circulation	Leaves	Boiled, drink the infusion
		Ornamental	Whole plant, by its beatiful leaves	Planted in private properties
		Medicinal (circulatory system) improve blood circulation	Leaves	Boiled, drimk the infusion
**SAURURACEAE**				
Anemopsis californica (Nutt.) Hook. & Anr., N, 24997	Hierba del manso	Medicinal (endocrin system), inflammation of lymph nodes; high temperature	Stems, leaves, inflorescences	Raw, milled, eat; boiled drink as tea
**SCROPHULARIACEAE**				
Leucophyllum frutescens (Berland.) I.M. Johnston, N, 25075	Cenizo	Medicinal (respiratory system), cough	Leaves	Boil, ingestion of infusion
		Ornamental	Whole plant, by its pereenial foliage and abundant flowers	Planted in private properties
		Medicinal (circulatory system), against	Leaves	Boiled, drink as a tea, several cups at day

		anemia		
		Medicinal (integumentary system), dermic infections	Branches and leaves	Toasted, apply as poultice in the affected area
Leucophyllum minus A. Gray, N, 25080	Cenizo	Medicinal (respiratory system), cough	Leaves	Boil, ingestion of infusion
**SELAGINELLACEAE**				
Selaginella lepidophylla (Hook. & Grev.) Spring, N, 24998	Flor de peña	Medicinal (digestive system), diarrhea	Leaves	Boiled, drink the infusion
**SOLANACEAE**				
Capsicum annuum L., N, 25043	Chile piquín	Condiment	Fruit	Raw or raw and milled, boiled with meal
		Medicinal (Respiratory system), cough	Fruit	Boiled, drink the infusion
		Medicinal (digestive system), hydration, hangover	Fruit	Mixed with meal, spicy food
		Medicinal (integumentaty system), extrneal wounds	Fruit	Boiled, the solution as poultice
Lycopersicon esculentum Mill., N, 25111	Tomate	Food	Fruit	Raw or cook
Solanum tuberosum L., E, 25079	Papa	Food	Root	Cook
Solanum rostratum Dunal, N, 25112	Mula	Medicinal (reproductive system), vaginal infections	Branches and leaves	Boiled, drink the infusion
**TURNERACEAE**				
Turnera diffusa Willd. ex Schult., N, 25076	Oreganillo	Medicinal (endocrine system), physical strenght	Leaves	Boiled, drink the infusion
		Medicinal (circulatory system), improve blood circulation	Leaves	Boiled, drink the infusion
		Medicinal (endocrin system), menopause	Whole plant	Boiled, drink the infusion
**URTICACEAE**				

Urtica chamaedryoides Pursh, N, 25077	Ortiga	Medicinal (lymphatic system), blood infections	Leaves and stems	Boiled, drink the infusion
**VERBENACEAE**				
Lippia graveolens Kunth, N, 25042	Orégano	Condiment	Leaves, flowers, and inflorescences	Dry, milled, add to the food
		Medicinal (respiratory system), eliminate phlegm	Leaves	Boiled, drink the infusion
**VITACEAE**				
Vitis vinífera L., E, 25078	Uva	Wine industry	Fruit	Smash, to produce wine
		Food	Fruit	Boiled to make jam
**XANTHORRHOEACEAE**				
Aloe vera L., E, 25041	Aloe, sábila	Medicinal (integumentary system), skin burns, epidermical wounds	Pulp	Raw, as poultice in the affected area
		Medicinal (digestive system), ulcer	Sap	Raw, drink
		Medicinal (integumentary system), scar	Leaves (pulp)	Raw, apply dirctly on the affected area
		Food	Leaves (pulp)	Milled, mixed with water, refreshing drink
**ZYGOPHYLLACEAE**				
Larrea tridentata (Sessé & Moc. ex DC.) Coville, N, 24999	Gobernadora	Medicinal (endocrine system), kidney diseases, remove kidney stones	Leaves	Boiled, put 5-6 leaves in a liter of boiling water for 20 seconds (it is very strong), drink as tea
		Medicinal (integumentary system), bad smell of feet	Leaves	Boiled, as poultice on the whole foot, also, put some dry leaves inside the shoes
		Medicinal (integumentary system), fungi on the feet	Branches	Boiled, apply as a poultice
		Medicinal	Branches	Boiled, apply

		(integumentary system), strengthen the hair		as shampoo directly on the hair
		Craft,	Branches	Boiled, the soluton used as tincture for saddles
		Furniture	Branches	To make chairs
		Medicinal (digestive system), to whet the apetite	Leaves	Boiled, 4-6 leves in wtaer, strong drink
		Medicinal (integumentary system), external wounds	Leaves	Boiled, apply as poultice int e affected area
**ZYNGIBERACEAE**				
Zingiber officinale Roscoe, E, 24984	Genjibre	Medicinal (endocrin system), physical weakness	Root	Boiled, drink the infusion
